# A Guard Cell Abscisic Acid (ABA) Network Model That Captures the Stomatal Resting State

**DOI:** 10.3389/fphys.2020.00927

**Published:** 2020-08-13

**Authors:** Parul Maheshwari, Sarah M. Assmann, Reka Albert

**Affiliations:** ^1^Department of Physics, Penn State University, University Park, PA, United States; ^2^Department of Biology, Penn State University, University Park, PA, United States

**Keywords:** stomatal closure, guard cell, Boolean network, Boolean model, memory, signal transduction

## Abstract

Stomatal pores play a central role in the control of carbon assimilation and plant water status. The guard cell pair that borders each pore integrates information from environmental and endogenous signals and accordingly swells or deflates, thereby increasing or decreasing the stomatal aperture. Prior research shows that there is a complex cellular network underlying this process. We have previously constructed a signal transduction network and a Boolean dynamic model describing stomatal closure in response to signals including the plant hormone abscisic acid (ABA), calcium or reactive oxygen species (ROS). Here, we improve the Boolean network model such that it captures the biologically expected response of the guard cell in the absence or following the removal of a closure-inducing signal such as ABA or external Ca^2+^. The expectation from the biological system is reversibility, i.e., the stomata should reopen after the closing signal is removed. We find that the model’s reversibility is obstructed by the previously assumed persistent activity of four nodes. By introducing time-dependent Boolean functions for these nodes, the model recapitulates stomatal reopening following the removal of a signal. The previous version of the model predicts ∼20% closure in the absence of any signal due to uncertainty regarding the initial conditions of multiple network nodes. We systematically test and adjust these initial conditions to find the minimally restrictive combinations that appropriately result in open stomata in the absence of a closure signal. We support these results by an analysis of the successive stabilization of feedback motifs in the network, illuminating the system’s dynamic progression toward the open or closed stomata state. This analysis particularly highlights the role of cytosolic calcium oscillations in causing and maintaining stomatal closure. Overall, we illustrate the strength of the Boolean network modeling framework to efficiently capture cellular phenotypes as emergent outcomes of intracellular biological processes.

## Introduction

Stomatal pores on the surfaces of leaves play an important role in allowing uptake of CO_2_ for photosynthesis and water vapor loss in transpiration. Guard cell pairs surround these stomatal pores and control their aperture by dynamic shrinking and swelling. Guard cells respond to numerous environmental signals, including light of different wavelengths and ambient CO_2_. In response to drought and other desiccating stresses, the plant produces the hormone abscisic acid (ABA), which induces stomatal closure. The process of stomatal closure in response to ABA involves the activity of many guard cell ion transport proteins, enzymes, and small molecules. There has been significant previous research involving experiments and theoretical and computational analyses to understand this complex process (Li et al., 2006; Ma et al., 2009; Sun et al., 2014).

Given the complex interactions among the many intracellular components of guard cells, network-based dynamic models constitute an efficient method for understanding the system. Dynamic models represent each intracellular component by a qualitative (discrete) or quantitative state variable, which describes the component’s abundance or activity. Dynamic models also describe how the interactions and regulatory relationships among components (represented as directed edges in the network) change each component’s state variable. Discrete dynamic models are simpler to create than quantitative models (which need extensive parameterization) yet they are rich enough to recapitulate and predict cellular behavior. The simplest type of discrete dynamic model is the Boolean model, wherein each node in the network is assumed to be either in the ON (1) or the OFF (0) state and each node state evolves in time according to its update function. The update function of a node is a logic function of the states of the regulators of the node and is expressed with the AND, OR, and NOT logic operators.

We have previously published network-based discrete dynamic models elucidating the process of stomatal response to various signals, including ABA and light of different wavelengths (Li et al., 2006; Sun et al., 2014; Gan and Albert, 2016; Albert et al., 2017; Maheshwari et al., 2019). These models use extensive iterations between experiments and model simulations. They capture the state corresponding to open or closed stomata as stable final states (attractors) of the dynamical system and they recapitulate almost all experimental results regarding knockout and constitutive activation of various nodes of the network. These models identify important mediators of stomatal closure induced by ABA or by external supply of mediators of ABA-induced closure (such as Ca^2+^). They also identify the various subnetworks that determine the different attractors of the network. These subnetworks, called stable motifs, form generalized positive feedback loops that once stabilized, maintain the constituent nodes in a fixed state (Zañudo and Albert, 2013). The most recent model (Maheshwari et al., 2019) identifies single nodes and combinations of nodes that are sufficient to drive the system to a particular attractor. The model also elucidates various crucial feedback loops that ensure the coordination between different components of the network.

The model of Maheshwari et al. (2019) however, does not recapitulate a few cases where the biological response is open stomata. In the model, stomatal closure is an attractor that is not reversible by the removal of the closure-inducing signal. In contrast, the biological reality in a situation of providing a closure-inducing signal for a limited period is gradual opening of the stomata following removal of the signal (Cummins et al., 1971). Furthermore, this model predicts ∼20% closed stomata in the absence of any signal (see Figure 3 of Maheshwari et al., 2019) while the biological reality is that the stomata remain open in the absence of any closure-inducing signal. Here, we present two different modifications of the network model of Maheshwari et al. (2019) such that it recapitulates the expected stomatal behavior in these situations. The first modification corresponds to revising certain assumptions of sustained activities for four nodes in the model. Instead of assuming persistent activity, we incorporate a short-term memory effect for these four nodes, where the node’s update function also considers the previous states of its regulator node. To understand the trajectories that lead to the open or closed stomata attractors, we construct the motif succession diagrams for the model version with persistent activity of these nodes as well as for the model version with short-term memory. The second modification corresponds to narrowing down the initial conditions used in the model simulations to ensure that the model recapitulates the open stomata state in the absence of any signal. This analysis elucidates the sensitive dependence of stomatal closure on the initial condition of six nodes whose pre-stimulus states are currently unknown. We use network analysis and causal logic (Maheshwari and Albert, 2017) to reveal the pathways by which these six nodes can lead to stomatal closure.

## Materials and Methods

### Background Information on the Boolean Model of ABA-Induced Stomatal Closure

Multiple iterations of experimental and computational research have led to a successful Boolean network model of ABA induced stomatal closure (Li et al., 2006; Albert et al., 2017; Maheshwari et al., 2019). The model relies on extensive literature curation to integrate the signaling components, interactions and mechanisms that underlie ABA-induced stomatal closure. The first version of the network model, containing 40 signaling components (Li et al., 2006), successfully recapitulated knockout phenotypes observed at that time and predicted many new phenotypes. One such prediction, regarding the importance of pH changes in ABA induced closure, was experimentally tested and validated in Li et al. (2006). The model later was expanded to 84 nodes based on new experimental results, for example concerning the identity of ABA receptors (Albert et al., 2017). The model was compared to a full complement of phenotypes observed by wet-bench experimentation and achieved a high degree of agreement. Several predictions of the model with regard to reactive oxygen species, cytosolic Ca^2+^, and heterotrimeric G-protein signaling were confirmed experimentally in Albert et al. (2017). For ease of simulation and understanding, this larger network model was later reduced to 49 signaling components in a way that preserves the outcomes of the model (Maheshwari et al., 2019).

**FIGURE 1:**
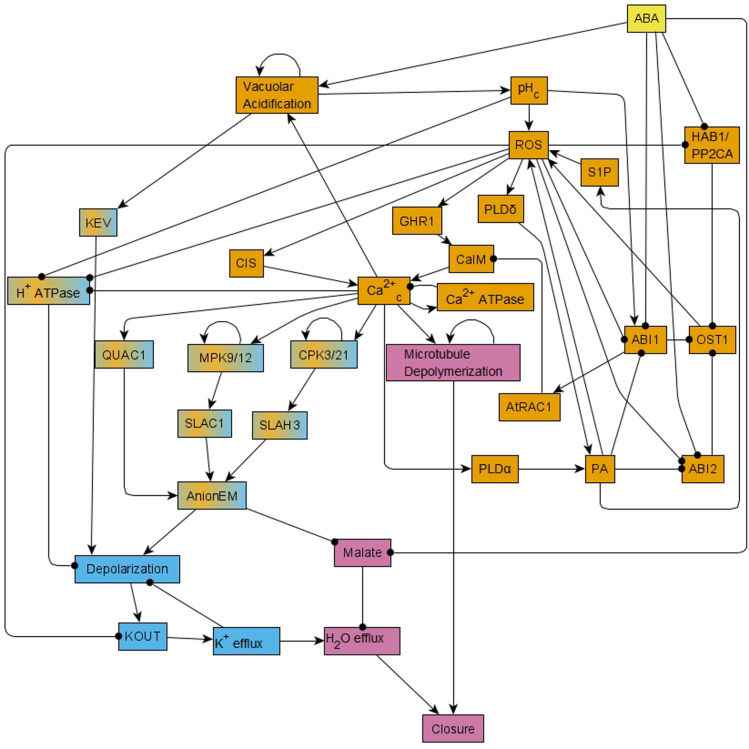
Simplified version of the network that forms the basis of the Boolean model of ABA induced stomatal closure. This network is reduced from the 49-node network in Maheshwari et al. (2019) using methods of causal logic reduction (Maheshwari and Albert, 2017) and binary transitive reduction (Albert et al., 2007); it preserves all the relationships among nodes of the 49-node network via edges or paths. Each edge that terminates in an arrow indicates an activating relationship and each edge that terminates in a black circle indicates an inhibitory relationship. This reduced network is presented here just for ease in visualization; all the analysis in this work was conducted on the 49-node network presented in Figure 1 of Maheshwari et al. (2019). This network contains positive feedback loops, i.e., cycles of directed edges that contain no or an even number of inhibitory edges, for example ROS – • ABI1 – • OST1→ ROS. It also contains negative feedback loops, i.e., cycles of directed edges that contain an odd number of inhibitory edges, for example Depolarization → KOUT → K^+^ efflux – • Depolarization. This network has two strongly connected components (SCCs) i.e., subnetworks in which every pair of nodes is connected by at least two paths of opposite direction. These two SCCs are represented in orange and light blue colors. Nodes of the orange SCC can reach the nodes of the light blue SCC via paths; the nodes linking these two SCCs are shown in a mix of orange and light blue colors. The signal ABA reaches all the nodes of the network. The out-component of this network leads from the two SCCs to the Closure node and is represented in pink color.

A significant insight obtained via this model was the recognition of a feedback-rich core that complements a canonical linear ABA signaling pathway. Sustained presence of ABA leads to ABA binding to RCAR/PYR1/PYL receptors (short for soluble pyrabactin resistance 1/pyrabactin resistance 1-like regulatory component of ABA receptor). This leads to the inhibition of the clade-A protein phosphatase 2Cs (PP2Cs), which relieves inhibition of the serine-threonine kinase OPEN STOMATA 1 (OST1). Kinase activity of OST1 results in reactive oxygen species (ROS) production, which in turn enhances Ca^2+^ uptake through the membrane (represented as the node CaIM in the model). This Ca^2+^ uptake, combined with Ca^2+^ release from intracellular stores (CIS), leads to cytosolic Ca^2+^ oscillations, which, together with the sustained presence of ABA, lead to production of phosphatidic acid (PA), ROS, and activation of phospholipase D delta (PLDδ). These three components form a positive feedback loop and hence they maintain their activation (Sierla et al., 2016). The cytosolic Ca^2+^ oscillations directly or indirectly also lead to activation of mitogen-activated protein kinases 9 and 12 (MPK9/12), calcium-dependent protein kinases 3 and 21 (CPK3/21), and depolymerization of microtubules. The ABA signal propagates through these various nodes and feedback loops to ultimately trigger opening of anion channels at the guard cell membrane (Kollist et al., 2014), leading to anion efflux. Anion efflux induces membrane depolarization, which in turn drives K^+^ efflux through depolarization-activated outward K^+^ channels (Ache et al., 2000; Hosy et al., 2003). Solute loss drives the osmotic efflux of water through aquaporins (Grondin et al., 2015), resulting in guard cell deflation and stomatal closure (Roelfsema and Hedrich, 2005). The 84-node network of ABA induced closure can be found as Figure 1 of R. Albert et al. (2017) and the reduced, 49-node version as Figure 1 in Maheshwari et al. (2019). In the version presented here in Figure 1, we incorporate further visual simplifications to ease understanding; we note that our analyses were done on the 49-node network of Maheshwari et al. (2019). The node names used in this paper are the abbreviated node names used in Maheshwari et al. (2019); the full names are indicated in Supplementary Table S1.

Multiple methods of analysis of the Boolean model have been used to understand the dynamics and outcomes of the system under different scenarios. One method is to specify the signal (usually, ABA) and the initial states of various components of the network and simulate the trajectory of the system over a given number of time-steps (∼50) using the software library BooleanNet (Albert et al., 2008). The initial condition corresponds to the information available on open stomata (see Supplementary Table S2). As there is no biological information available from the experimental literature about the pre-stimulus state of multiple nodes [there are 17 such nodes in the 49-node model of Maheshwari et al. (2019)], these nodes were started in a randomly selected initial condition. At each time-step, the nodes of the system are updated (i.e., their states are recalculated) in a randomly selected order (Wang et al., 2012). This is an appropriate update scheme for the stomatal closure network since the timescales of the internal processes are largely unknown. During each simulation, each node in the network changes state one or multiple times and after sufficient time, the system settles down into an attractor. Generally, an attractor can be a fixed point (steady state) or a set of states that repeat indefinitely (a complex attractor, corresponding to a sustained oscillation). The Boolean model of ABA-induced closure yields a complex attractor in which most of the nodes (40 out of 49) have a stationary state. Due to the stochasticity introduced by the update method and by the initial node states, a large number (∼2000) of replicate simulations are run. The outcome of the model is summarized as the percentage of simulations in which the node Closure is in the state 1 (ON) at each time step, which we refer to as the percentage of closure. To characterize a whole time-course of closure in response to ABA or another signal, the cumulative percentage of closure (CPC) is defined as the sum of the percentage of closure over the course of the simulation (usually 50 time steps). The external supply or constitutive activation of a node is implemented as a sustained ON state, the knockout of a node as a sustained OFF state, and the time-course of a thus-perturbed system is compared to that of the wild-type system (see Section “Simulation of Node Constitutive Activation”).

A useful analytical tool for obtaining the system trajectories and attractors of the ABA network is to identify stable motifs, which are generalized positive feedback loops that maintain an associated state of their constituent nodes (see Section “Stable Motifs and Oscillating Motifs” and Zañudo and Albert, 2013). For example, the positive feedback between PA, ROS and PLDδ determines a stable motif that ensures the sustained ON state of these nodes. Each stable motif can yield the stabilization of further nodes in the network, trapping the system’s state into a subset of the state space. One can follow an iterative procedure where the influence of each stable motif is used to reduce the network and one then finds the stable motifs in the reduced network. Some of these secondary stable motifs are dependent on certain conditions such as the prior stabilization of a node. For this reason, they are called conditionally stable motifs (see Section “Stable Motifs and Oscillating Motifs” and Deritei et al., 2019). The ABA-induced closure network also contains a negative feedback loop formed by the nodes Ca^2+^_*c*_ and Ca^2+^ATPase (see Figure 1), which under certain conditions (e.g., in the presence of ABA) induces the sustained oscillation of these nodes and of a few nodes regulated by them. This negative feedback loop is an example of a conditional oscillating motif (see Section “Stable Motifs and Oscillating Motifs”).

Starting from a signal and obtaining the consecutive (conditionally) stable motifs and (conditional) oscillating motifs gives us the motif succession diagram, which reflects the system’s trajectories and identifies the system’s attractors (see Section “Stable Motif Succession Diagrams of the Stomatal Closure Model Versions”). Another complementary approach is to use causal logic analysis to find long-range causal dependencies (i.e., sufficient or necessary subgraphs) or cyclic causal dependencies, which correspond to stable motifs (Maheshwari and Albert, 2017). This method was used to identify the logic backbone of the ABA-induced closure process. For example, ABA is sufficient for the activation of the node pH_*c*_ (meaning an increase in the cytosolic pH level) and also sufficient for deactivation of the AtRAC1 protein (Maheshwari and Albert, 2017). Causal logic analysis can also be used to find the causal effect of a node perturbation and to identify certain interventions that can independently drive the network into a certain attractor. For example, causal logic indicates that the sustained ON state of the node ROS is a driver of a stable motif corresponding to stomatal closure in the absence of ABA (Maheshwari and Albert, 2017). Indeed, experimental results demonstrate that providing H_2_O_2_ leads to stomatal closure (Zhang et al., 2001; Kwak et al., 2003).

Overall, the results in Albert et al. (2017) illustrate a comprehensive process of model construction from causal relationships, model analysis through simulations and network-based methods, model validation, and use of the model to make novel predictions. Most of the discrepancies between model results in Albert et al. (2017) and experimental data involve the model’s inability to recapitulate closure in response to the constitutive activation of a node that is causally sufficient for Ca^2+^_*c*_ oscillations. This observation inspired the prediction of a possible Ca^2+^ regulation of PP2C activity. Following a systematic analysis, Maheshwari et al. (2019) confirm, both *in silico* and experimentally, that Ca^2+^ directly or indirectly (via the activation of PA production) inhibits the PP2C ABSCISIC ACID INSENSITIVE 2 (ABI2). The thus-updated model resolves the previous discrepancies. The analysis in Maheshwari et al. (2019) also identifies the stable motif associated with stomatal closure in the absence of ABA, the drivers of this motif, and the causal relationships between various non-canonical closure signals. The basis of our present analysis is the two versions of the model reported in Maheshwari et al. (2019), which we will refer to as Model1 and Model2. The difference between the two models is that in Model1 PA is assumed to inhibit ABI2 while in Model2 Ca^2+^_*c*_ is assumed to inhibit ABI2.

### Methods

#### Boolean Update Functions

In a Boolean model the future state of a node is determined by the current state of its upstream regulators and is expressed as a Boolean update function. In the following we will use a simple notation convention for Boolean update functions: represent the state of each node by the name of the node and mark the future state with an asterisk. A node with a single regulator is characterized by one of two types of single variable Boolean functions: identity and negation. Identity is used for positive regulators. Denoting the regulator (and its state) by X and the target node by Y, the Boolean update function of Y is Y^∗^ = X, meaning that the target Y is adopting the state of the regulator X as its future state. Negation, expressed using the Boolean operator “not,” is used for negative regulators. The Boolean update function of Y is Y^∗^ = not X, meaning that the target Y adopts the opposite state as the regulator. Notice that in both cases the future state of Y depends only on the state of its regulator X.

For nodes that have more than one regulator, the “or” operator is used if a particular state of any of the regulators can independently activate the target; the “and” operator is used if a particular state of each of the regulators is needed for activation. The choice of the most appropriate operator to use is determined by experimental evidence. Our analysis uses the Boolean update functions constructed in Albert et al. (2017), and then simplified in Maheshwari et al. (2019). Supplementary Table S3 indicates the simplified update functions of each node (as in Maheshwari et al., 2019) and experimental literature supporting it.

#### Including Memory in Boolean Update Functions

In most of the Boolean update functions used in models of biological systems the regulators of the target node do not include the node itself, which means that the future state of the node does not depend on its current state. In contrast to differential equation-based models, for which the change in a molecule’s concentration is described by explicit synthesis term(s) and degradation term(s) (Tyson and Novak, 2020), the degradation in Boolean models is implicit. For example, the update function Y^∗^ = X implies that the off state of X triggers the off state of Y regardless of whether Y was on before. In cases where there is a known mechanism for positive self-regulation of a target node and/or there is evidence that the target node’s activity is longer-lived than the activity of its activators, it is justified to include the target node as its own self-activator. Applying this modification to the previous example yields X^∗^ = Y or X, which implies that X will stay in the on state after achieving it for the first time.

There were four such cases in the model of ABA-induced closure (Albert et al., 2017; Maheshwari et al., 2019), namely CPK3/21, MPK9/12, vacuolar acidification and the depolymerization of microtubules. All four of these nodes are activated by an increase in the cytosolic calcium level (Ca^2+^_*c*_). However, Ca^2+^_*c*_ elevation cannot be maintained indefinitely because it would lead to precipitation of calcium-phosphates, which would be toxic to the cell. It is also well-documented that Ca^2+^_*c*_ oscillates during the process of stomatal closure (Staxén et al., 1999). Yet, there is no evidence that these four nodes would have an oscillatory pattern, or decrease when the cytosolic Ca^2+^_*c*_ level decreases, during ABA induced stomatal closure. On the contrary, their sustained activation is necessary to achieve sustained anion efflux and stomatal closure. Thus, in Albert et al. (2017) it was assumed that the activity of these four nodes decays very slowly or not at all. This assumption was supported by a specific mechanism in case of three of the four nodes (see Supplementary Text S1). The assumption was implemented by including the state of the node in its own update function, connected by the “or” operator to the state of Ca^2+^_*c*_. For example, the update function of CPK3/21 is CPK3/21^∗^ = Ca^2+^_*c*_ or CPK3/21. According to this function, the next state of CPK3/21 equals 1 (ON) if Ca^2+^_*c*_ is currently 1, or if the current state of CPK3/21 is 1. Once CPK3/21 turns on, it will stay on the whole duration of validity of the model, which represents the 30−60 min needed to achieve closure in response to ABA. We refer to this assumption as the assumed persistent activity of these four nodes.

Here, we study a longer process that consists of stomatal closure induced by ABA, then reopening following the removal of the signal. We therefore introduce an alternative implementation of slow decay of the activity of these four nodes: we include past states of Ca^2+^_*c*_ in their update function. For example, CPK3/21^∗^ = Ca^2+^_*c (t)*_ or Ca^2+^_*c (t–*__1__)_ or Ca^2+^_*c (t–*__2__)_ where t is the current time step. This function indicates that the next state of CPK3/21 equals 1 if Ca^2+^_*c*_ is currently ON or has been ON in the last two time steps. We refer to this implementation as short-term memory. The duration of the short-term memory (in units of a time step) is an adjustable parameter.

### Simulation of Node Constitutive Activation

The constitutive activation (CA) of a node is simulated by setting its state to ON and keeping it fixed to ON throughout the entire simulation – this can be thought of as equivalent to providing a biomolecule in non-limiting quantity or, for an enzyme, providing the enzyme in non-limiting quantity and in the active state, during an experiment. These *in silico* mutations are categorized into different response categories (defined in Maheshwari et al., 2019) by their steady state percentage of closure and by their cumulative percentage of closure (CPC) values. In the simulations in the absence of ABA, we refer to the scenario where no node is kept constitutively active as the baseline. Significantly increased response is the case when the percentage of closure reaches 100%. Slightly increased response is the case when the final percentage of closure is higher than the baseline but less than 100%. Close to baseline is the case when the percentage of closure and CPC are within two standard deviations of the respective baseline values. As described in Section “Recapitulating the Open Stomatal State in the Absence of a Signal,” baseline percentages of closure can be non-zero.

### Evaluation of Consistency Between Simulation and Experiment

We group the experimental response categories into two broad classes: close to baseline response, which indicates a stomatal aperture that is not statistically significantly different from wild type in the absence of a signal, and increased response (decreased aperture compared to wild type). Slightly or significantly increased response compared to baseline are considered consistent with experimental observation of increased response (decreased aperture compared to the wild type) in the absence of ABA. Close to baseline response is considered consistent with a stomatal aperture measured in the absence of ABA that is not statistically significantly different from or is greater than that of the wild type.

### Stable Motifs and Oscillating Motifs

A stable motif of a Boolean dynamical system is a generalized feedback loop that maintains an associated state of its constituent nodes regardless of the state of nodes outside of the feedback loop. Stable motifs were first defined using the expanded network, a network that encodes the causal relationships between node states (as incorporated in the update functions). The expanded network consists of two virtual nodes for each node (one for each of the two possible states) and composite nodes that embody AND gates among two or more node states. A stable motif is a subgraph of an expanded network that satisfies four properties: (1) it is strongly connected (there is a path between every pair of nodes in the subgraph), (2) it is consistent (all represented states can be simultaneously satisfied), (3) it is composite-closed (if a composite node is in the subgraph, so too are all its virtual node parents), and (4) it is minimal (it contains no non-trivial subgraphs satisfying the first three properties). Stable motifs can also be defined using the causal logic formalism of Maheshwari and Albert (2017) i.e., a cyclic sufficient subgraph, necessary subgraph, or both sufficient and necessary subgraph determines a stable motif. After the nodes of a stable motif adopt the associated state, they will stay in that state. This may induce other nodes to adopt fixed states as well. Thus, a stable motif determines a region of the state space from which dynamical trajectories cannot escape. In this so-called trap space, a subset of the nodes have a fixed state; for this reason this trap space is also referred to as a partial fixed point.

Another important class of expanded network subgraph is the oscillating motif. Like stable motifs, oscillating motifs are strongly connected, composite-closed subgraphs of the expanded network. Unlike stable motifs, however, oscillating motifs violate the consistency criterion in that every virtual node in the subgraph has its negation in the subgraph as well. Intuitively, oscillating motifs arise from negative feedback loops, while stable motifs arise from positive feedback loops. An oscillating motif will likely (but not always) correspond to a complex attractor in which the nodes represented in the oscillating motif oscillate.

A weaker version of stable motifs was defined by Deritei et al. (2019) and named conditionally stable motif. A conditionally stable motif is a consistent, strongly connected component of the expanded network that is not composite closed. The virtual nodes that are parent nodes of composite nodes included in the conditionally stable motif serve as the conditions. In other words, the conditionally stable motif is a generalized positive feedback loop that maintains an associated state as long as one or more nodes outside of the feedback loop are in a specific state. As long as these parent nodes are in that specific state, the conditionally stable motif behaves like a stable motif. Conditional oscillating motifs can be defined analogously as strongly connected, consistency-violating, not composite-closed subgraphs of the expanded network.

### Motif Succession Diagram Analysis

In general, the stable motif succession diagram is determined by following these steps. First, the system’s stable motifs are determined. For every stable motif, the corresponding node states are substituted into the Boolean regulatory functions and the network is iteratively reduced. Then, the stable motifs of the reduced network are determined; these may be stable motifs or conditionally stable motifs of the original network. This process is repeated until the network cannot be reduced anymore. Nodes remaining in the network may oscillate in the attractor(s), while the nodes that were reduced due to the influence of a stable motif will take the associated fixed values. The stable motif succession diagram encapsulates all successions of stable motifs found by this process. For more details, see Zañudo and Albert (2013), where a Java implementation is also provided.

In this paper, we perform motif succession diagram analysis for two assumptions regarding the nodes CPK3/21, MPK9/12, Microtubule Depolymerization and Vacuolar Acidification. The first assumption is persistence of these four nodes, as described in Section “Including Memory in Boolean Update Functions.” The second assumption is short term memory for these four nodes; their corresponding motif succession diagrams are detailed in Section “Stable Motif Succession Diagram Analysis of the Network with Short-term Memory.” We generate the stable motif succession diagrams for these networks primarily using the Stable Motif code provided in Zañudo and Albert (2013) and supplementing those results by theoretical analysis. The different signal conditions are set by setting the state of the signal node (e.g., ABA or Ca^2+^) to ON or OFF and reducing the network with that setting. We augmented these diagrams with our analysis of each conditional oscillatory motif, including identification of its condition and of the nodes whose state stabilizes due to the establishment of this motif. We use the causal logic framework from Maheshwari and Albert (2017) to aid in this analysis.

## Results

### Stable Motif Succession Diagrams of the Stomatal Closure Model Versions

As described in Section “Including Memory in Boolean Update Functions” and Supplementary Text S1, the original model of ABA-induced closure (Albert et al., 2017; Maheshwari et al., 2019) includes the assumption that the activity of four nodes regulated by the cytosolic calcium level (Ca^2+^_*c*_), namely CPK3/21, MPK9/12, Vacuolar Acidification and Microtubule Depolymerization, decays very slowly or not at all. The assumption was implemented by introducing their current state in the update function of these nodes. For example, the update function of CPK3/21 is CPK3/21^∗^ = Ca^2+^_*c*_ or CPK3/21. The assumed persistence of the activity of CPK3/21 constitutes a stable motif: once activated, CPK3/21 maintains its ON state regardless of the rest of the nodes. An analogous stable motif also exists for each of the other three nodes. These stable motifs appear as self-loops on the four nodes in Figure 1. We determined the succession diagrams of stable and oscillating motifs in three cases: absence of ABA, presence of ABA, and externally provided Ca^2+^. In this section, we present examples of stable motif succession diagrams of Model1 and describe the changes observed in Model2.

#### ABA OFF Case

As reported in Maheshwari et al. (2019), in the absence of ABA there are 17 attractors. Of these attractors, only one, denoted A0, corresponds to closed stomata. The attractors A1 to A16 are highly similar and are consistent with open stomata (see Supplementary Table S4). These attractors differ in the state of a few nodes that can be stabilized in either the ON or OFF state or can oscillate, while still corresponding to the biologically known information on open stomata.

**FIGURE 2:**
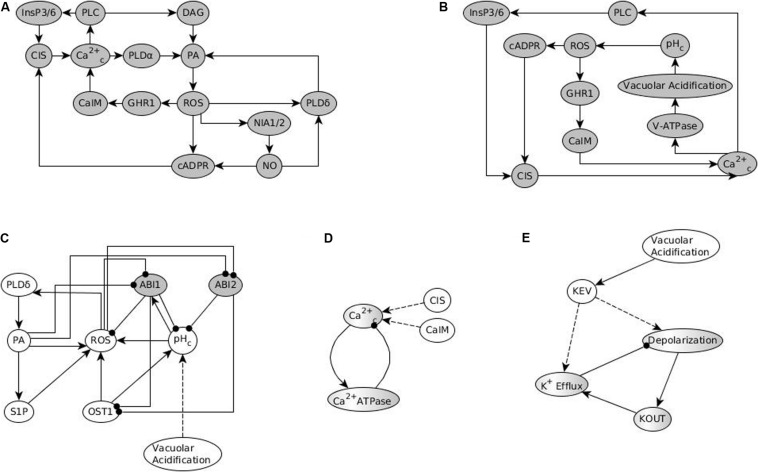
Stable and oscillating motifs observed in the absence of ABA. **(A)** Stable motif associated with open stomata attractors; we refer to this motif as *openM1.* **(B)** Stable motif associated with open stomata attractors; we refer to this motif as *openM2*. **(C)** Conditionally stable motif associated with the closed stomata attractor A0; we refer to this motif as *closureM*. The condition for this conditionally stable motif is the ON state of Vacuolar Acidification. In panels **(A–C)**, the white background indicates the ON state of the node and the gray background means OFF state of the node. **(D)** Conditional oscillatory motif associated with closed stomata. This motif plays a role in both the presence and absence of ABA. The condition for the activation of this motif is the ON state of either of CIS or CaIM, and it yields a sustained oscillation of Ca^2+^_*c*_ and Ca^2+^ ATPase (represented as a gray-white background). **(E)** Conditional oscillatory motif, *K*^+^ *oscillation*, that exists in the absence of any signal. The condition for the activation of this motif is KEV = ON. Since Vacuolar Acidification is sufficient for KEV, the ON state of Vacuolar Acidification is sufficient to establish this conditional oscillating motif, leading to sustained oscillation of its constituent nodes.

The network in the ABA = 0 case has 6 stable motifs, 4 of which correspond to the persistent ON states of CPK3/21, MPK9/12, Microtubule Depolymerization, and Vacuolar Acidification. The remaining 2 stable motifs, which we denote *openM1* and *openM2*, represent the simultaneous OFF state of more than 10 nodes (see Figures 2A,B).

There also are 5 conditionally stable motifs (CSMs). Four of these represent the persistent OFF state of each of the four self-regulating nodes. The OFF state of CPK3/21, MPK9/12, and Microtubule Depolymerization are conditioned on either *openM1* or *openM2*. The OFF state of Vacuolar Acidification is conditioned on *openM1* (it is part of *openM2*). The fifth CSM, shown in Figure 1C, expresses the self-sustained activity of PLDδ, PA, ROS, S1P, OST1, and pH_*c*_ together with the sustained inactivity of ABI1 and ABI2. This CSM is dependent on the condition that the Vacuolar Acidification = 1 stable motif is activated. There are two conditional oscillating motifs. The first is made up by the nodes Ca^2+^_*c*_ and Ca^2+^ATPase, both of which oscillate as long as CIS = 1 or CaIM = 1. The second motif comprises the oscillations of the K^+^ efflux, KOUT and Depolarization nodes, conditioned on KEV = 1. We refer to this latter motif as *K^+^ oscillation*. These motifs are presented in Figure 2.

The stabilization of the CSM shown in Figure 1C ensures the establishment of sustained Ca^2+^_*c*_ oscillations. Indeed, if either of the positive regulators of Ca^2+^_*c*_ is stabilized in its active state, the negative feedback between Ca^2+^_*c*_ and Ca^2+^ATPase makes both of them oscillate (as characterized in detail in Supplementary Text S2 of Maheshwari et al., 2019). The CSM shown in Figure 1C includes the sustained ON state of the node ROS, which is sufficient for the node CIS. The CSM also includes the sustained OFF state of ABI2, which in combination with the ON state of ROS is sufficient for the node CaIM. Thus, the stabilization of the CSM leads to Ca^2+^_*c*_ – Ca^2+^ ATPase oscillations. As a result of these oscillations, most nodes directly downstream of Ca^2+^_*c*_, i.e., PLC, PLDδ, QUAC1, TCTP, V-ATPase, DAG, and InsP3/6, also oscillate. Their oscillation periods are given in Supplementary Table S4 and explained in Maheshwari et al. (2019). The remaining two nodes directly regulated by Ca^2+^_*c*_, namely CPK3/21 and MPK9/12, persist in their ON state after first turning on. The oscillations of TCTP stabilize the Microtubule Depolymerization = 1 motif. Altogether, the stabilization of the CSM leads indirectly to the sustained ON state of Microtubule Depolymerization (via sustained Ca^2+^_*c*_ oscillation), and to the sustained ON state of H_2_O Efflux (via the sustained ON state of multiple nodes downstream of the CSM). The activation of H_2_O Efflux and Microtubule Depolymerization, in turn, leads to stomatal closure. Since this CSM drives the network into an attractor corresponding to closed stomata (see Supplementary Table S4), we will refer to it as *closureM.*

**FIGURE 3:**
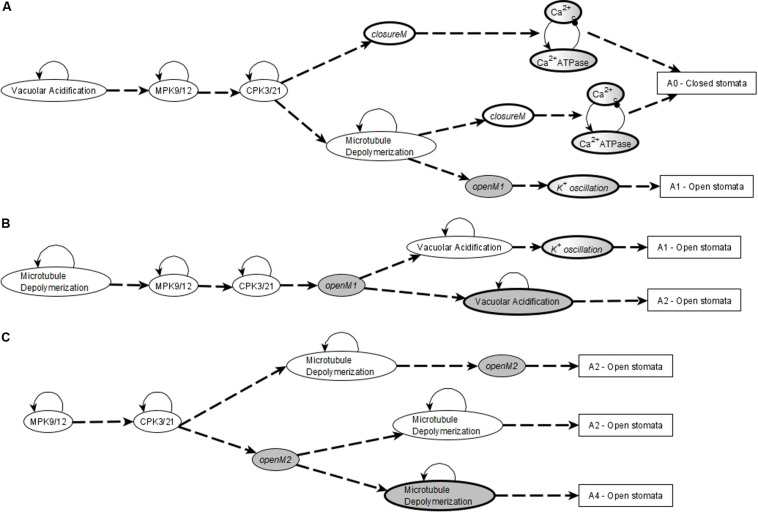
Motif succession diagrams of Model1 in the absence of ABA and any other closure signal. Stable motifs are shown with oval symbols and attractors are indicated by rectangles. Each dashed directed line between two motifs indicates that the system states in which the first motif has established and any nodes driven by it have stabilized admit the second motif as next to activate. The dashed directed line converging into an attractor symbol indicates that the succession of stable motifs ensures the system’s convergence into the respective attractor. **(A)** shows a subset of the succession diagram that converges to the attractor A0, corresponding to closure of the stomata in the absence of ABA (see Supplementary Table S4). The stable motif characteristic to this attractor is the conditionally stable motif *closureM*. **(B,C)** show a subset of the succession diagram corresponding to a sample of the 16 attractors that describe open stomata in the absence of ABA (see Supplementary Table S4). Each possible trajectory in this case contains exactly one of the stable motifs *openM1* and *openM2*. **(B)** describes some of the trajectories containing *openM1* while C describes some of the trajectories containing *openM2.* **(A)** also indicates the existence of bifurcations in the system’s trajectory due to the mutually exclusive motifs *closureM* and *openM1.* If *closureM* stabilizes the system converges into the attractor A0, and if *openM1* stabilizes the system converges into attractor A1. The diagram encodes node states into the background color of the stable motif symbols. When referring to single nodes, white background indicates the ON state of the node, gray background means the OFF state of the node, and gray-white background represents oscillating nodes. Since in the *openM1* and *openM2* stable motifs all nodes are OFF (see Figure 2), we use a gray background color for these stable motifs. We use white background to represent the locking in of the *closureM* motif and represent the oscillating nature of the *K^+^ oscillation* motif by a gray-white background. The conditionally stable motifs are marked by thick boundaries.

In conclusion, any system trajectory that (i) involves the stabilization of the stable motifs Vacuolar Acidification = ON, CPK3/21 = ON, MPK9/12 = ON, Microtubule Depolymerization = ON, *closureM*, and the Ca^2+^_*c*_ – Ca^2+^ ATPase conditional oscillating motif, and (ii) satisfies the restrictions that Vacuolar Acidification = ON establishes before *closureM* and *closureM* stabilizes before the Ca^2+^_*c*_ – Ca^2+^ ATPase motif, reaches the closure attractor A0. Figure 3A shows two of the possible trajectories to the closed stomata attractor A0 when the first motif to stabilize is Vacuolar Acidification = ON, the second motif to stabilize is MPK9/12 = ON and the third motif to stabilize is CPK3/21 = ON. Figure 3A also illustrates that if the Vacuolar Acidification stable motif is already locked in then the system’s trajectories may bifurcate. In one branch the *closure* motif stabilizes, leading to the closure attractor A0. In the other branch the *openM1* stable motif stabilizes, which leads to the open stomata attractor A1.

Figure 3B denotes two possible motif successions that contain the stable motif *openM1* and lead to an attractor that corresponds to open stomata. In both successions, the first motif to stabilize is Microtubule Depolymerization = ON, the second motif to stabilize is MPK9/12 = ON, and the third motif is CPK3/21. *openM1* is the condition for the CSMs Vacuolar Acidification = OFF, CPK3/21 = OFF, MPK9/12 = OFF, and Microtubule Depolymerization = OFF. Hence, a possible system trajectory involving *openM1* can have both the OFF state and ON state of the four self-regulating nodes after *openM1* is established, while it can have only their ON state before *openM1*. Any trajectory that contains *openM1* and the Vacuolar Acidification = ON motif leads to the establishment of the conditional oscillating motif *K^+^ oscillation*. These cases give rise to open stomata attractors that have the K^+^ efflux, KOUT and Depolarization nodes oscillating. Trajectories that contain *openM1* and the Vacuolar Acidification = OFF motif lead to open stomata attractors in which K^+^ efflux, KOUT and Depolarization are off. As a result, this case has many possible trajectories and hence leads to a complex succession diagram, one branch of which is displayed in Figure 3B.

Figure 3C denotes a possible succession diagram leading to an open stomata attractor that involve the stabilization of *openM2*. The Vacuolar Acidification = ON motif is mutually exclusive with *openM2*. Similar to the case of *openM1*, the OFF states of CPK3/21, MPK9/12 and Microtubule Depolymerization are CSMs with *openM2* as the condition. Hence, a possible system trajectory involving *openM2* can have either the ON or OFF state of the three self-regulating nodes after *openM2* but it can have only their ON state before *openM2*. Figure 3C shows the case when the first motif to stabilize is MPK9/12 = ON and the second is CPK3/21.

#### ABA ON Case

**FIGURE 4:**
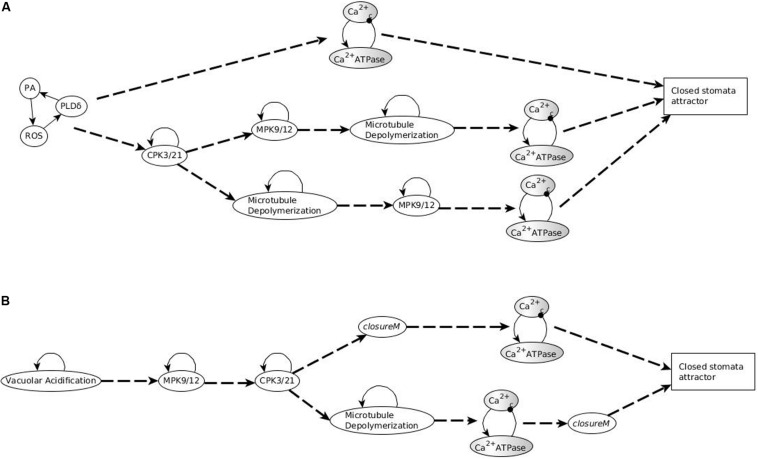
Motif succession diagram of Model1 in the presence of a closure inducing signal. As in Figure 3, stable motifs are shown with oval symbols and attractors are indicated by rectangles. Each dashed directed line between two motifs indicates that the system states in which the first motif has established and any nodes driven by it have stabilized admit the second motif as next to activate. The dashed directed line converging into an attractor symbol indicates that the succession of stable motifs ensures the system’s convergence into the respective attractor. White background indicates the ON state of the corresponding node and white-gray background indicates oscillating nodes. **(A)** Motif succession diagram in the sustained presence of ABA when the first motif to stabilize is the PA = 1, PLDδ = 1, ROS = 1 stable motif. The top trajectory is the case when the oscillatory motif stabilizes into oscillations after the PA-PLDδ-ROS motif stabilizes; as a result of the Ca^2+^_*c*_ – Ca^2+^ ATPase oscillations, the motifs CPK3/21 = 1, MPK9/12 = 1, and Microtubule Depolymerization = 1 stabilize directly and hence the system stabilizes in the closed stomata attractor. The middle and bottom trajectories represent the case when the second motif to stabilize is CPK3/21 = 1. The complete succession diagram covers all possible trajectories that start with stabilization of one or more of the four stable motifs (in any order), followed by the activation of the oscillatory motif, after which the system always stabilizes into the closed stomata attractor. **(B)** Motif succession diagram when external Ca^2+^ is simulated as the fixed ON state of the CaIM node when the first three motifs to stabilize are Vacuolar Acidification = ON, MPK9/12 = ON, and CPK3/21 = ON.

In the presence of ABA, the network has four stable motifs. Three of these express the self-sustained activity of CPK3/21, MPK9/12, and Microtubule Depolymerization, respectively; these were stable motifs in the absence of ABA as well. The self-loop of Vacuolar Acidification does not appear as a stable motif because the ON state of Vacuolar Acidification is indirectly determined by ABA and does not need self-stabilization. The fourth motif is a cycle that sustains the ON state of PLDδ, PA, and ROS. This cycle is a subset of the *closureM* motif; the rest of the nodes of this motif are stabilized by ABA. There is also an oscillatory motif comprised of Ca^2+^_*c*_ and Ca^2+^ ATPase. It is the same as the conditional oscillating motif in the ABA = OFF case, however, in the ABA = ON case, it is not conditional anymore, because the sustained presence of ABA leads to the ON state of CaIM, which fulfills the condition for Ca^2+^_*c*_ – Ca^2+^ ATPase oscillations. The stabilization of these four motifs, in any order, leads to the sole attractor reachable in this case. As expected, and consistent with the knowledge that ABA is sufficient for stomatal closure, this attractor corresponds to closed stomata. It differs from the closure attractor A0 reached in the absence of ABA in the state of five nodes whose state is determined solely by ABA: RCARs, V-PPase, Actin reorganization (which are ON in the presence of ABA and OFF in its absence) and PEPC, AtRAC1 (which are OFF in the presence of ABA and ON in its absence). A subset of the motif succession diagram for this case is presented in Figure 4A.

#### Externally Provided Ca^2+^ as Signal

In the absence of ABA, external Ca^2+^ can be simulated either as the fixed ON state of the Ca^2+^_*c*_ node or as the fixed ON state of the CaIM node, which represents calcium influx through the membrane. In the case of fixed ON state of the Ca^2+^_*c*_ node the stable motifs in the network corresponding to closed stomata (i.e., the four self-regulating nodes and the *closureM* motif) are quickly stabilized, because Ca^2+^_*c*_ is a driver node for all of them. Thus, any initial condition leads to an attractor corresponding to closed stomata. This attractor is slightly different from the attractor corresponding to closure induced by ABA in that there are no oscillating nodes (see Supplementary Table S4). In fact, the effect of fixed ON state of Ca^2+^_*c*_ is slightly stronger than the effect of the sustained presence of ABA, which leads to sustained Ca^2+^_*c*_ oscillations. This is because the fixed ON state of Ca^2+^_*c*_ drives the network into a closed stomata attractor in fewer time steps than the sustained presence of ABA.

When the external Ca^2+^ is simulated as the fixed ON state of the CaIM node, the network has all of the motifs associated with the closed stomata attractor in the absence of ABA, i.e., the four stable motifs corresponding to persistent activity, the *ClosureM* motif and the oscillatory motif containing Ca^2+^_*c*_ and Ca^2+^ ATPase (whose condition is now satisfied). The succession diagram of this case, shown in Figure 4B, is very similar to the one shown in Figure 3A except that the Ca^2+^_*c*_ – Ca^2+^ ATPase oscillating motif is not a conditional motif anymore and can hence form a trajectory by appearing in any order with the four stable motifs. The *closureM* motif is still a conditionally stable motif. Figures 3B,C are not possible in this case since the ON state of CaIM is incompatible with the *openM1* and *openM2* motifs. In other words, the only attractor possible in this case is the closed stomata attractor A0.

The stable motifs and the succession diagram of Model2, i.e., the network model in which Ca^2+^_*c*_ directly inhibits ABI2, are the same as those of Model1 in the presence of ABA and in the presence of external calcium. There are some small differences between the models in the absence of ABA, which are described in Supplementary Text S2.

### Relaxation to Resting State After Removing the Signal

**FIGURE 5:**
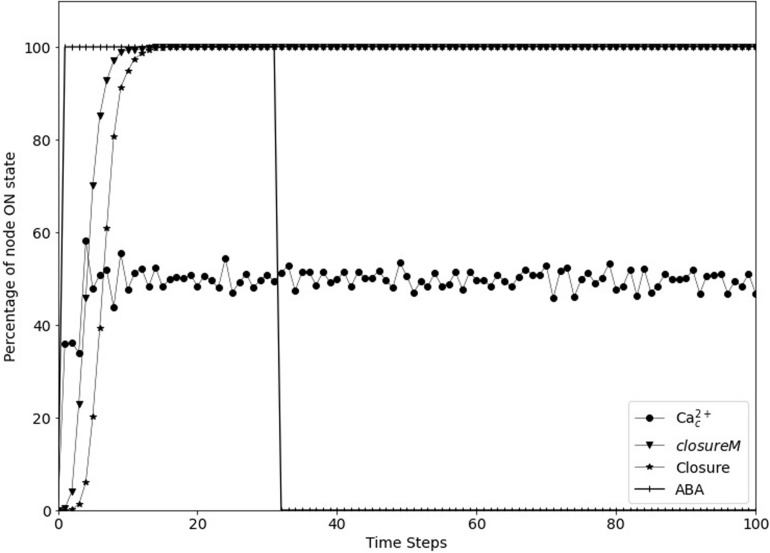
Simulated stomatal closure is maintained after removal of ABA. The state of the node ABA (vertical line symbols) is ON for 30 time-steps and is then OFF. The *closureM* conditionally stable motif (downward pointing triangles) establishes in less than 15 timesteps and remains stable despite the loss of ABA. The percentage of closure (star symbols) increases to 100% and stays at this value even after the signal is removed. The circles represent the percentage of the ON state of Ca^2+^_*c*_ which after a fast increase fluctuates around 50% since Ca^2+^_*c*_ oscillates with approximately equal ON and OFF time periods. The biological expectation is that after the signal is removed the states of ROS, Closure, and Ca^2+^_*c*_ should go to OFF eventually.

According to our analysis of the model in Maheshwari et al. (2019), stomatal closure involves the stabilization of four stable motifs, the *ClosureM* conditionally stable motif (whose condition is the Vacuolar Acidification stable motif) and a Ca^2+^_*c*_ − Ca^2+^ATPase conditional oscillating motif (whose condition is satisfied by the *ClosureM* motif, see Section “Stable Motif Succession Diagrams of the Stomatal Closure Model Versions”). The four stable motifs stay in their associated state once stabilized. Even if the signal is taken away in the model, these stable motifs still stay stabilized, and so do the conditional motifs (since their condition is still satisfied), keeping the simulated stomata closed. Figure 5 shows a simulation of Model1, indicating that removal of the signal (ABA) does not lead to re-opening of the simulated stomata. This is not accurate since the biological reality is the reopening of the stomata after ABA is removed (Cummins et al., 1971). Here, we resolve this discrepancy by making the activity of certain nodes less persistent than assumed in Albert et al. (2017) and Maheshwari et al. (2019).

**FIGURE 6:**
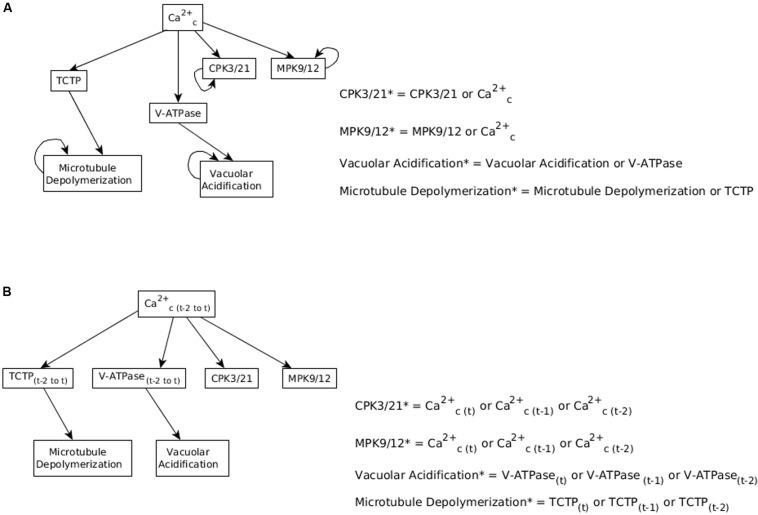
Considering memory of the states of regulator nodes instead of persistent activity. **(A)** In Maheshwari et al. (2019), the nodes CPK3/21, MPK9/12, Vacuolar Acidification, and Microtubule Depolymerization have a persistence term in their update function to maintain them in a fixed ON state when Ca^2+^_*c*_ oscillates. **(B)** Replacing persistent activity with short-term memory. The update function of each of the four nodes combines with an “OR” function the states of their respective regulator (Ca^2+^_*c*_, TCTP, or V-ATPase) at the current and previous two time-steps.

As described in Section “Including Memory in Boolean Update Functions,” the model currently assumes persistent activity of CPK3/21, MPK9/12, Microtubule Depolymerization and Vacuolar Acidification, which form stable motifs (see Figure 3). These stable motifs make the closure attractor irreversible, which is not biologically accurate. Hence, we considered the possibility of decreasing the persistence of these nodes, i.e., we assumed that the state of the target node is sustained only for a few time steps after its regulator (e.g., Ca^2+^_*c*_) turns off, after which the target node goes back to the OFF state. We implement this short-term memory by storing the state of the regulator in the past few timesteps (see Figure 6). Specifically, we use auxiliary nodes to remember the past states of the regulator (e.g., Ca^2+^_*c*_); these auxiliary nodes are always updated before the rest of the nodes are updated.

**FIGURE 7:**
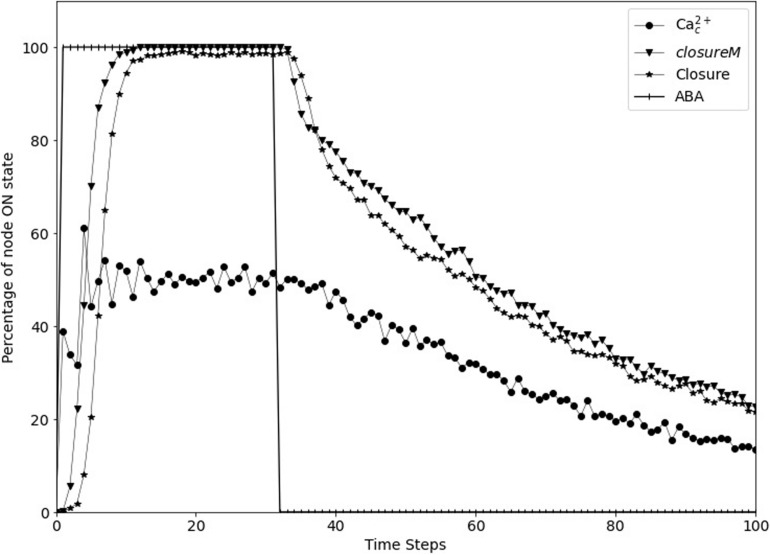
Stomatal re-opening after removal of the signal in the updated Model1. The signal, ABA (vertical lines) is set to fixed ON state for the first 30 time-steps and then set to fixed OFF state for 70 time-steps. The *closureM* conditionally stable motif (downward triangles) is stabilized within the first 10 time-steps and it slowly destabilizes after ABA is set to the OFF state. The state of the node Closure, depicted by the star symbols, shows a similar behavior i.e., it starts decreasing after the signal is removed. The state of the node Ca^2+^_*c*_, depicted by the circle symbols, is oscillating in the presence of ABA and it slowly transitions to the OFF state after ABA is fixed to OFF state.

We use the ranked asynchronous update of the BooleanNet software library, in which the nodes are classified as rank 1, 2, … and during each timestep, nodes of rank 1 are updated first (according to a random order among these nodes), followed by nodes of rank 2, and so on. We designate the auxiliary nodes corresponding to the largest memory as rank 1, the auxiliary nodes with next largest memory as rank 2, and so on. The regular nodes of the network have the lowest rank (highest numerical value). For example, when Microtubule Depolymerization considers the last five timesteps of TCPT, CPK3/21 and MPK 9/12 consider the last three timesteps of Ca^2+^_*c*_ and Vacuolar Acidification considers the last two timesteps of V-ATPase (as in Figure 7), the auxiliary nodes TCTP_5, Ca_3 and V-ATPAse_2 have rank 1, the auxiliary nodes TCTP_4, Ca_2 and V-ATPase_1 have rank 2, the auxiliary nodes TCTP_3 and Ca_1 have rank 3, the auxiliary node TCTP_4 has rank 4, the auxiliary node TCTP_5 has rank 5 and all the regular nodes have rank 6. The Boolean update functions for this case are given in Supplementary Text S3.

We implemented short-term memory effect for the four nodes and explored various durations for each of them to identify cases which show efficient closure in response to ABA and efficient re-opening of the stomata after ABA is removed. The analysis of these durations, described in Supplementary Text S4, indicates that a memory of three timesteps ensures the persistent ON state of CPK3/21 and MPK9/12, while the persistence of Microtubule Depolymerization or Vacuolar Acidification would be ensured for a memory of six timesteps. We found that the best result is exhibited by the model version where Vacuolar Acidification considers the last 2 time-steps of V-ATPase, CPK3/21 and MPK9/12 each consider the last 3 time-steps of Ca^2+^_*c*_, and Microtubule Depolymerization considers the last 5 time-steps of TCTP. Figure 7 shows the re-opening of stomata after the removal of ABA with these parameters. The assumed memory effect is weakest for the node Vacuolar Acidification; for two timesteps memory the probability of the ON state of this node is 85% (see Supplementary Text S4). This node is required to be in the ON state for the stabilization of the *closureM* motif and the turning off of this node contributes to reopening of the stomata. The larger memory duration of three timesteps ensures the persistence of CPK3/21 and MPK9/12 activity. The five timestep memory of Microtubule Depolymerization yields a 97% probability of its ON state. Overall, these memory durations ensure close to 100% closure in response to ABA, followed by reopening after the loss of the ABA signal.

### Stable Motif Succession Diagram Analysis of the Network With Short-Term Memory

**FIGURE 8:**
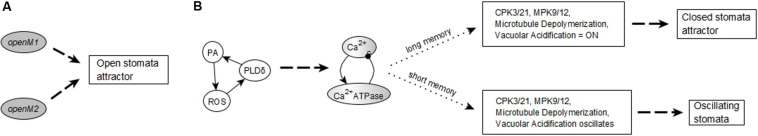
Succession diagram for Model1 with short term memory. **(A)** Stable motif succession diagram in the absence of ABA and any other closure-inducing signal. Regardless of the duration of the memory, this case always leads to an open stomata attractor. **(B)** Motif succession diagram in the presence of ABA. The system can reach two different attractors depending on whether the memory duration is large enough. When the memory duration is large enough, sustained Ca^2+^_*c*_ oscillations can sustain the ON state of the nodes CPK3/21, MPK9/12, Microtubule Depolymerization, and Vacuolar Acidification. With small memory duration these nodes oscillate instead of stabilizing; as a result, the node corresponding to stomatal closure also oscillates. See Supplementary Text S3 for details on the sufficient memory durations and Supplementary Table S4 for these attractors. The dashed edges denote logic succession with certainty while the dotted edges denote the variant outcomes depending on the memory duration.

In the updated model with short term memory, the nodes CPK3/21, MPK9/12, Microtubule Depolymerization and Vacuolar Acidification do not form stable motifs anymore. Indeed, their future state is strictly independent of their own current state and is solely determined by their regulators’ current and past states. Since these nodes no longer form motifs, the stable motif succession diagram is modified. However, the short-term memory effect needs to be strong enough to ensure that in the presence of a signal (for example, ABA or external calcium) there is a system trajectory in the succession diagram that leads to the closed stomata attractor. This is possible as ABA or external calcium causes Ca^2+^_*c*_ oscillations, which are sufficient to maintain these four nodes in their ON state if the memory duration is sufficiently large (see Supplementary Text S4 and Figure 8).

#### ABA OFF Case

In the absence of ABA, similar to the case described in Section “Stable Motif Succession Diagrams of the Stomatal Closure Model Versions,” Model1 has two stable motifs, *openM1* and *openM2*; these motifs are given in Figure 2. The stabilization of either of these two stable motifs causes a fixed OFF state of Ca^2+^_*c*_, which in turn leads to a fixed OFF state of CPK3/21, MPK9/12, Microtubule Depolymerization and Vacuolar Acidification. This leads to an open stomata attractor (see Supplementary Table S4). The presence of only two motifs leads to the possibility of just two trajectories, depicted in Figure 8A.

#### ABA ON Case

In the presence of ABA, Model1 has two motifs, one of which is a stable motif comprised of PA = 1, PLDδ = 1 and, ROS = 1, and other is the oscillating motif consisting of Ca^2+^_*c*_ and Ca^2+^ ATPase. This oscillating motif is the same as the one described in the ABA = ON case in Section “Stable Motif Succession Diagrams of the Stomatal Closure Model Versions,” and as in that case the condition of this motif is satisfied since ABA indirectly activates CaIM. The sustained oscillations of Ca^2+^_*c*_ are sufficient to fix the ON state of CPK3/21 and MPK9/12 if their memory is three or more time steps; they will fix Microtubule Depolymerization and Vacuolar Acidification if their memory is six or more time steps (see Supplementary Text S4). Stabilization of these two motifs (in either order) leads to the closed stomata attractor when the memory duration is large enough (see Figure 8B). If the memory duration is smaller, the system instead converges into an attractor where many nodes, including Closure, oscillate (see Supplementary Table S4). After ABA is removed, the condition for the conditional oscillating motif of Ca^2+^_*c*_ and Ca^2+^ ATPase is no longer satisfied. Hence, the oscillating motif ceases to exist and the Ca^2+^_*c*_ oscillations decay, which leads to the fixed OFF state of CPK3/21, MPK9/12, Microtubule Depolymerization and Vacuolar Acidification. This in turn leads to an open stomata attractor (see Supplementary Table S4).

#### Externally Provided Ca^2+^ as the Signal

Similar to Section “Stable Motif Succession Diagrams of the Stomatal Closure Model Versions,” we considered two methods of simulating external Ca^2+^. With Ca^2+^_*c*_ fixed in the ON state, the states of CPK3/21, MPK9/12, Microtubule Depolymerization and Vacuolar Acidification are fixed in the ON state too. This leads to activation of the *closureM* motif, as described in Section “Relaxation to Resting State After Removing the Signal,” and hence the network stabilizes in a closed stomata attractor. When external Ca^2+^ is simulated as fixed ON state of CaIM, the Ca^2+^_*c*_ – Ca^2+^ ATPase oscillating motif establishes. Indeed, experiments confirm that high external Ca^2+^ leads to sustained oscillations in Ca^2+^_*c*_ (Jeon et al., 2019). The sustained oscillations of Ca^2+^_*c*_ in the model lead to sustained ON state of CPK3/21, MPK9/12 if their memory is three or more time steps, and to sustained ON state of Microtubule Depolymerization and Vacuolar Acidification if their memory is six or more time steps. The sustained ON state of Vacuolar Acidification leads to establishment of the *closureM* motif. The percentage of simulations in which the *closureM* motif stabilizes is always less than the percentage of ON state of Vacuolar Acidification and it increases as the memory duration is increased – see Supplementary Figure S7. Once the *closureM* motif stabilizes, it leads the system to the closed stomata attractor.

The stable motifs and the succession diagram of Model2 with the short-term memory effect are the same as those of Model1 in the presence of ABA or in the presence of external calcium. Similar to the case described in Section “Stable Motif Succession Diagrams of the Stomatal Closure Model Versions,” there are some small differences between the models in the absence of ABA, which are described in Supplementary Text S2.

### Recapitulating the Open Stomatal State in the Absence of a Signal

**FIGURE 9:**
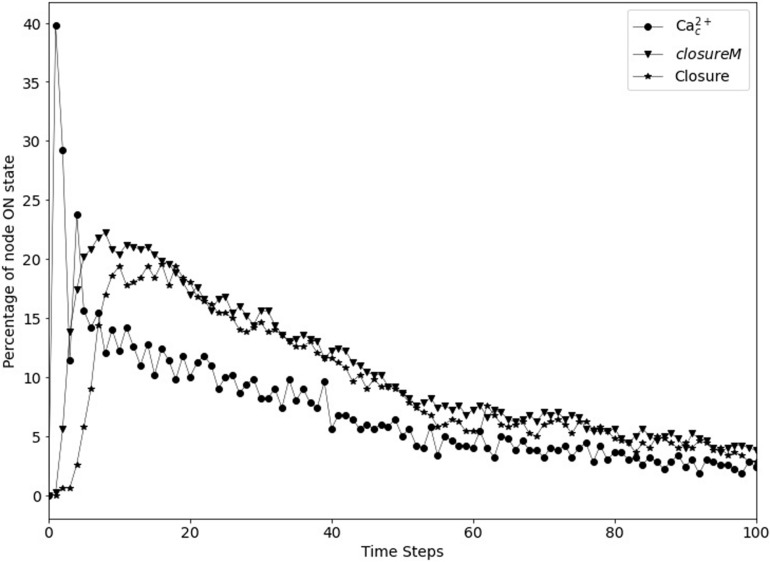
Transient closure observed in Model1 in the absence of ABA when using short-term memory instead of node persistence. Due to the random initial conditions of the 17 nodes, the *closureM* motif temporarily stabilizes in ∼29% of the simulations (downward triangles) and there is a non-zero level of Ca^2+^_*c*_ oscillations, which in turn lead to up to ∼20% transient closure. Eventually, the percentage of closure reduces to less than 5%.

The model in Maheshwari et al. (2019) yields ∼20% closed stomata in simulated wild type guard cells that did not receive any closure signal. We refer to the cumulative percentage of closure (CPC) obtained in the simulation of unstimulated wild type cells as baseline. The biological expectation is that the baseline percentage of closure should be 0. We determined the baseline percentage of closure in the updated Model1 that has short term memory instead of persistent activity of CPK3/21, MPK9/12, Microtubule Depolymerization, and Vacuolar Acidification. We found that the percentage of closure approaches 0 after a long time; however, there is a non-zero transient level of closure (see Figure 9), and thus a non-zero baseline CPC. While this is a significant improvement compared to the final percentage of ∼20% reported in Maheshwari et al. (2019) and further supports the replacement of persistent activity with short-term memory, this is still not an accurate recapitulation of the biological expectation. Hence, in this section, we explore ways to achieve zero cumulative percentage of closure to better recapitulate the biological expectation.

In Maheshwari et al. (2019), seventeen nodes of the network were initialized randomly because of the lack of experimental evidence regarding their state in open stomata. These nodes are indicated in Supplementary Table S2. We reported in Maheshwari et al. (2019) that the non-zero baseline percentage of stomatal closure can be attributed to the initial condition. When certain nodes are initialized randomly and by chance their initial state is the same as their state in the closed stomata attractor, then the initial state essentially acts as a transient (single timestep) closure signal, which may in certain cases lead to the activation of the *closureM* motif. As we see in Figure 7, after a closure signal is removed, the percentage of closure, activation of the *closureM* motif, and Ca^2+^_*c*_ oscillations reduce over time. We observe a similar behavior in Figure 9 where, as a result of the initial condition the percentage of closure, motif stabilization and Ca^2+^_*c*_ oscillations initially increase, and then reduce to less than 5% after 100 timesteps. The peak of transient percentage of closure, motif stabilization or Ca^2+^_*c*_ oscillations is much lower in Figure 9 than Figure 7 because the probability of activation of the *ClosureM* motif due to the initial condition is much lower than the probability of activation due to an ABA signal sustained for 30 timesteps. Hence, we hypothesized that the model would yield zero cumulative percentage of closure (CPC) if all of these seventeen nodes were initiated in the state opposite to their state corresponding to closed stomata. This “furthest from closure” initial condition indeed resulted in zero CPC in both Model1 and Model 2 (that is, both when PA inhibits ABI2 and when Ca^2+^_*c*_ is assumed to directly inhibit ABI2).

**FIGURE 10:**
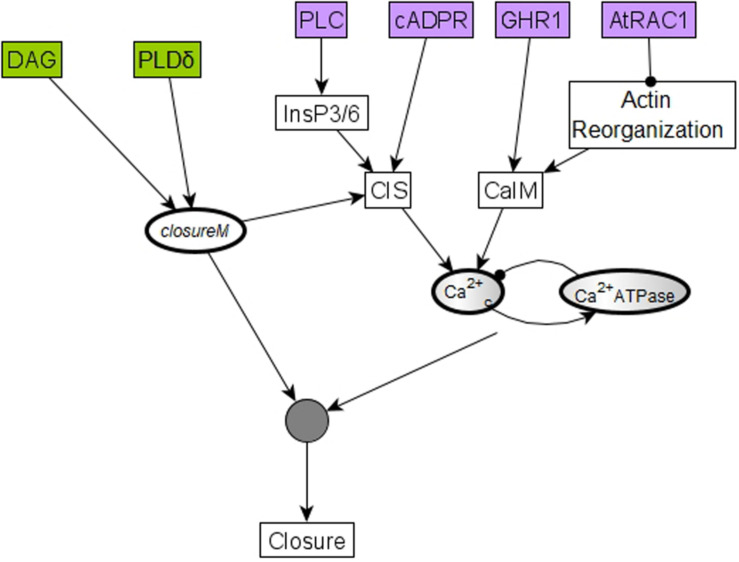
The initial condition of six nodes can lead to temporary closure by helping establish Ca^2+^_*c*_ oscillations and the *closureM* conditionally stable motif. Four of these six nodes, cADPR, GHR1, AtRAC1, and PLC, represented in purple, regulate one of the two processes (CIS or CaIM) that yield Ca^2+^_*c*_ elevation. For example, the two-edge path between AtRAC1 and CaIM indicates that AtRAC1 inhibits the reorganization of the actin cytoskeleton that otherwise would contribute to Ca^2+^ influx (CaIM). Ca^2+^_*c*_ elevation, in turn, has some probability of causing Ca^2+^_*c*_ oscillations due to the negative feedback loop formed by Ca^2+^_*c*_ and Ca^2+^_*c*_ ATPase. These oscillations have a potential to drive the network to the closed stomata attractor. The remaining two nodes, PLDδ and DAG, represented in green, affect the *closureM* motif, indicated by the edges that start from PLDδ and DAG, respectively, and end in the node that stands for the *closureM* motif. Both nodes are direct regulators of PA, which is an internal driver of the motif, thus their activity has a chance of locking in the *closureM* motif. The stabilization of the *closureM* motif is sufficient for sustained Ca^2+^_*c*_ oscillations, indicated by the path mediated by CIS. The cumulative effect of sustained Ca^2+^_*c*_ oscillations and of the *closureM* motif leads to stomatal closure (see Section “Stable Motif Succession Diagrams of the Stomatal Closure Model Versions”). Hence, initiating any of these six nodes in their states corresponding to stomatal closure leads to a non-zero percentage of stomatal closure, at least transiently.

The “furthest from closure” initial condition assumes a particular state for each of the 17 nodes that were initialized randomly (Maheshwari et al., 2019). Since the pre-stimulus state of none of these nodes was measured experimentally, the validity of each of these assumptions is unknown. To reduce the chance of incorrect assumptions, we next explore the possibility of minimizing the number of nodes for which such assumptions are made while still ensuring a zero baseline CPC. We use logical analysis and simulations to find this minimal restriction on initial conditions. In Model1, i.e., the model version where PA inhibits ABI2 through an edge, we find that in order to obtain a baseline CPC of zero, the initial states of 6 of the 17 randomly initialized nodes need to be the opposite of their state corresponding to closed stomata. These six nodes and their corresponding states are cADPR = OFF, GHR1 = OFF, AtRAC1 = ON, PLC = OFF, PLDδ = OFF, and DAG = OFF. All of these nodes affect the *closureM* conditionally stable motif directly or indirectly. The nodes PLDδ and DAG are sufficient activators of the node PA, which is an internal driver node of *closureM* (if Vacuolar Acidification has already stabilized in the ON state). The remaining four nodes, i.e., cADPR, GHR1, AtRAC1, and PLC, promote either Ca^2+^ influx through the membrane (CaIM) or Ca^2+^ release from intracellular stores (CIS), hence promoting Ca^2+^_*c*_ oscillations – see the purple nodes in Figure 10. Ca^2+^_*c*_ is an external driver of *closureM* (Maheshwari et al., 2019), as it can induce the ON state of the Vacuolar Acidification node (the condition of the *closureM* motif) and induce the ON state of PA, the ON state of pH_*c*_ and the OFF state of ABI2. Since these 3 nodes form a 3-node driver of this conditionally stable motif, the ON state of Ca^2+^_*c*_ leads to stabilization of *closureM* (Maheshwari et al., 2019). Sustained oscillations of Ca^2+^_*c*_ can also lead to the stabilization of the *closureM* motif if the relationship between the Ca^2+^_*c*_ = ON period and the short-term memory of Vacuolar Acidification is such that the Vacuolar Acidification = ON condition is satisfied (see Maheshwari et al., 2019, Supplementary Text S3, and Supplementary Figure S7). Hence if any of these four nodes are initialized in their state corresponding to stomatal closure, there is a chance of indirectly stabilizing the *closureM* motif and to transiently driving the system to closure.

In Model2, i.e., the model version where the inhibition of ABI2 happens through a direct edge from Ca^2+^_*c*_, we found that a baseline CPC of zero is achieved when the initial state of just four of the 17 nodes is specified and the remaining 13 are initialized randomly. These four nodes are shown in purple background in Figure 10; their corresponding states are cADPR = OFF, GHR1 = OFF, AtRAC1 = ON and PLC = OFF. These four nodes are a subset of the six nodes we identify in the case of Model1; specifically, they are the nodes that can promote Ca^2+^_*c*_ oscillations. Since in Model2 Ca^2+^_*c*_ directly inhibits ABI2, the initial conditions that promote Ca^2+^_*c*_ have a slightly higher likelihood of leading to closure. In Model2 PA is not an internal driver of the *closureM* motif (Maheshwari et al., 2019), thus the initial state of nodes DAG or PLDδ does not play a role in promoting the stabilization of this motif and hence promoting closure. In summary, Model2 requires fewer restrictions than Model1 to avoid the activation, in the absence of ABA, of the *closureM* conditionally stable motif.

We evaluated the agreement of these model versions, sampling their respective sets of initial conditions that correspond to zero baseline closure percentage, with the extant experimental evidence for the effects of constitutive activation of nodes (e.g., whether constitutive activation of a node induces a significant decrease in stomatal aperture, see Section “Evaluation of Consistency Between Simulation and Experiment”). The model-indicated effects of constitutive activation of single nodes fall into three categories: close to baseline response (which in this case means a CPC of zero), slightly increased response, and significantly increased response (which in this case leads to a final closure percentage of 100%). In Model1, i.e., the model version where PA directly inhibits ABI2, the simulations agree with experimental observations in 14 instances and they disagree in 3 instances (see Table 1 and Supplementary Table S5). The instances of agreement include six cases in which the model and experiments agree in observing closure (decreased stomatal aperture) in case of constitutive activation of the corresponding node and 8 cases in which neither the model nor experiments observe closure. The instances of agreement include the constitutive activation of CaIM, which happens in experiments where Ca^2+^ is provided externally (see last entry of Table 1). The experimental observation of Ca^2+^_*c*_ oscillations, as well as stomatal closure, in response to the presence of external Ca^2+^ (Jeon et al., 2019) also supports the model prediction that the sustained Ca^2+^_*c*_ oscillations are sufficient for stomatal closure.

**TABLE 1:** Comparison between experimental results and simulation results in Model1 with short-term memory for constitutive activation of nodes in the absence of ABA.

**Node that is constitutively active**	***In silico* response when using the initial condition furthest from closure**	***In silico* response when using the least restricted initial condition**	**Experimentally observed response**
TCTP	Close to baseline	Close to baseline	Close to baseline (Kim et al., 2012)
ROP11	Close to baseline	Close to baseline	Close to baseline (Li et al., 2012)
Microtubule Depolymerization	Close to baseline	Close to baseline	Close to baseline (Jiang et al., 2014)
PLDα	Close to baseline	Slightly increased	Close to baseline (Mishra et al., 2006)
PA	Close to baseline	Slightly increased	Increased (Jacob et al., 1999)
NO	Close to baseline	Slightly increased	Increased (Desikan et al., 2002)
S1P	Close to baseline	Close to baseline	Increased (Ng et al., 2001; Coursol et al., 2003)
AtRAC1	Close to baseline	Close to baseline	Close to baseline (Lemichez et al., 2001)
H^+^ ATPase	Close to baseline	Close to baseline	Close to baseline (Wang et al., 2014)
PP2CA	Close to baseline	Close to baseline	Close to baseline (Kuhn et al., 2006)
ABI1	Close to baseline	Close to baseline	Close to baseline (Allen et al., 1999)
ABI2	Close to baseline	Close to baseline	Close to baseline (Allen et al., 1999)
cADPR	Significantly increased	Significantly increased	Increased (Joudoi et al., 2013)
InsP3/6	Significantly increased	Significantly increased	Increased (Gilroy et al., 1990)
ROS	Significantly increased	Significantly increased	Increased (Zhang et al., 2001; Kwak et al., 2003)
CaIM	Significantly increased	Significantly increased	Increased (Jeon et al., 2019)

*We performed 500 simulations over 50 time-steps in each setting. The cumulative percentage of closure (CPC) for the WT simulation (where no node was constitutively activated) was 0.0. Each row of the table indicates the node that is constitutively active, the response category obtained from the model for two different initial conditions, and the experimentally observed response. There are three response categories: close to baseline, slightly increased compared to baseline, and significantly increased compared to baseline (see Section “Evaluation of Consistency Between Simulation and Experiment”). The second column corresponds to the furthest from closure initial condition, where the 17 nodes with unknown pre-stimulus state are initialized in the opposite of the state they achieve in the closure attractor. The third column corresponds to the least restricted initial condition that gives zero baseline closure percentage and CPC, in which six nodes, namely cADPR, GHR1, AtRAC1, PLC, PLDδ, DAG are initiated in the opposite of the state they achieve in the closure attractor and 11 nodes are initialized randomly. The fourth column lists the experimentally observed responses for each of the node constitutive activation with the corresponding literature references. The experimental responses have two categories: close to baseline and increased response (i.e., decreased aperture). When using the furthest from closure initial condition there are 13 cases of agreement with experiments and 4 cases of disagreement. When using the least restricted initial condition there are 14 cases of agreement and 3 cases of disagreement. All the differences between results lie in the constitutive activation of PLDα, PA, S1P and NO. Supplementary Table S6 indicates the CPC values of each case.*

Conversely, Model1 yields a zero percentage of closure in case of constitutive activity of S1P and pH_*c*_ (as neither S1P nor pH_*c*_ can independently stabilize the *closureM* motif) while a decreased aperture was observed experimentally. In case of PLDα constitutive activity Model1 yields slightly higher than baseline closure (as PLDα leads to PA production, which is an internal driver of the *closureM* motif) while experiments did not observe a statistically significant decrease in stomatal aperture. In the “furthest from closure” initial condition (when nodes are initialized in the state opposite their state corresponding to stomatal closure) there are 13 cases of agreement and four cases of disagreement. The 13 cases of agreement include four cases of significantly increased closure and nine cases of close to baseline closure. In addition to S1P and pH_*c*_, there is also disagreement for external supply of PA and NO, where the model yields close to baseline response but a higher than baseline degree of closure was observed experimentally. Similar to the case of S1P and pH_*c*_, PA and NO cannot independently stabilize the *closureM* motif. They can however stabilize the motif in a fraction of cases when initial conditions are favorable.

In Model2, i.e., the model version where Ca^2+^_*c*_ directly inhibits ABI2, we found the same instances of agreement (13) and disagreement (4) as for the “furthest from closure” initial conditions of Model1 (see Supplementary Table S6). The reason for the cases of disagreement between experimental results and simulation results remains the same as for Model1: none of S1P, NO, PA, or pH_*c*_ can stabilize the *closureM* motif. Each of them can only activate the motif as part of a two- or three-node driver set (e.g., the two-node driver set of PA and Vacuolar Acidification and the three-node driver set of S1P, Vacuolar Acidification, and PLDδ). The same categories of responses to node constitutive activations, and thus the same cases of disagreement, were found for the “furthest from closure” initial condition and for the least restrictive initial condition that yields a baseline percentage of closure of zero.

We next sought to determine whether initiating one or more nodes of the previously restricted sets, i.e., (cADPR, GHR1, AtRAC1, PLC) or (cADPR, GHR1, AtRAC1, PLC, PLDδ, DAG), respectively, in a random state would still maintain near-zero baseline closure while increasing the percentage of closure when S1P, NO, PA or pH_*c*_ is constitutively activated. We indeed found this to be the case. A few slightly less restricted initial conditions in Model1 lead to a non-zero but small value of transient baseline closure (see Supplementary Table S7). These cases show improved agreement with experiments regarding node constitutive activation compared to the least restricted initial condition summarized in column 2 of Supplementary Table S6. Specifically, the constitutive activation of pH_*c*_ is now in the slightly increased category, in agreement with experimental results, while its categorization in Supplementary Table S6 as yielding close to baseline response disagreed with experiments. The node pH_*c*_ alone is not sufficient to be a driver of the *closureM* motif; however, it can contribute to 2-node or 3-node drivers when combined with the initial activity of DAG or PLDδ. When the system starts in an initial state that includes DAG or active PLDδ there is a non-zero probability of the *closureM* motif stabilizing and leading to the closed stomata attractor.

In Model2, we found initial conditions where the simulations of node constitutive activations include fewer cases of disagreement with experimental results than the initial conditions that ensure a CPC of zero, but these initial conditions also result in a CPC that is significantly higher than zero. For example, random initialization of PLC yields a higher than baseline closure in case of constitutive activation of each of S1P, PA, and pH_*c*,_ with the only remaining disagreement being NO. The trade-off for this regained agreement of the simulated node constitutive activation with experiments is an increase in the baseline percentage of closure and CPC: the peak percentage of closure is 3% and the CPC is 1.91. In conclusion, it is not possible to simultaneously ensure a zero baseline percentage of closure and recapitulate closure in response to constitutive activity of S1P or pH_*c*_ given our current knowledge of the biological resting/open stomata state. The node initializations that help achieve better agreement with experimental data on node constitutive activations also increase the baseline percentage of closure.

## Discussion

We present ways to improve the Boolean model of ABA-induced stomatal closure by Maheshwari et al. (2019) to recapitulate biological expectations regarding stomata remaining in an open state in the absence of closure signals, or relaxing back to the open stomata state following the loss of closure signals. We find that modifying the assumed persistent activity of four nodes to a short-term memory effect helps recapitulate re-opening of the stomata after the closure signal is removed. The implementation of such short-term memory still yields transient closure in the absence of any closure signal; thus we also identify different combinations of initial conditions that minimize this transient closure. We find that the percentage of stomatal closure is sensitive to the initial states of certain nodes. This highlights the significance of these internal nodes and the importance of experimentally determining the resting states (or open stomata states) of these nodes.

Motivated by the incorporation of timing in Boolean modeling in Thakar et al. (2007), we replaced the assumed persistent activity of four nodes, i.e., CPK3/21, MPK9/12, Microtubule Depolymerization, and Vacuolar Acidification, with a function that considers the cumulative effect of the current and the past states of their regulator. We show that this short-term memory effect of an oscillating regulator, for example, Ca^2+^_*c*_, helps these four nodes maintain the persistent activity necessary to ensure stomatal closure in response to closure-inducing signals. This assumption brings the model closer to biological reality by exhibiting reopening of the stomata after the closure-inducing signal is removed. We perform a systematic study of the effect of varying memory durations on the extent of stomatal closure and the rate of reopening upon removal of the signal. Our analysis of the increase in the percentage of ON state of a node for larger memory duration can be extended to other patterns of oscillations of the regulator node and hence it will be useful in various other Boolean models of biological networks.

Our analysis demonstrates that motif succession diagrams provide a powerful means to present and understand the system trajectories, highlighting the points of no return in the system’s dynamics and identifying the various attractors the system can lead to. In this work, we present the motif succession diagram of the stomatal closure network, which integrates and summarizes previous research on this network (Li et al., 2006; Albert et al., 2017; Maheshwari et al., 2019). This succession diagram highlights the key role of oscillating motifs, drawing attention to the significance of oscillations in this network. This advances our understanding of node oscillations in the context of a biological system modeled as a Boolean network. Seeking motivation from recent work (Deritei et al., 2019), we also identify conditionally stable and conditional oscillating motifs and differentiate them from their condition-free counterparts.

The motif succession diagram is also an effective measure of the consequences of short-term versus long-term memory. Comparing Figure 8A with Figure 3, we can see that short-term memory eliminates the trajectories that would yield a closure attractor in the absence of ABA and reduces the variability of the open stomatal attractors. Another way to illustrate the qualitative difference between short-term and long-term memory is through a bifurcation diagram (Tyson and Novak, 2020), which indicates the steady state value(s) of the node Closure for different values of ABA. As described in Supplementary Text S5, an on-off-on sequence of ABA yields an irreversible switch in the status of closure in case of persistent activity of CPK3/21, MPK9/12, Microtubule Depolymerization and Vacuolar Acidification, while short-term memory of the same nodes yields reversible closure. Comparing Figure 8B with Figure 4, we can see that short-term memory leads to an attractor in which the Closure node oscillates in the presence of ABA. The fact that oscillating stomatal apertures have not been observed experimentally in response to ABA suggests that the biological persistence of vacuolar acidification, microtubule depolymerization and of the activity of CPK3/21 and MPK9/12 is essential to the control of oscillations. This also highlights the importance of characterizing and quantifying the biological mechanisms underlying the persistence of these nodes (see Supplementary Text S1).

This work and our previous research on stable motifs (Zañudo and Albert, 2013; Steinway et al., 2014; Albert et al., 2017; Zanudo and Albert, 2015; Maheshwari et al., 2019; Rozum and Albert, 2018; Gan and Albert, 2018; Deritei et al., 2019) contributes to the broader field of investigation that connects positive feedback loops, multistability, cell fates and phenotypes (Thomas and Ari, 1990; Huang, 2007; Hari et al., 2020). Specifically, single or intersecting positive feedback loops form stable motifs or conditionally stable motifs. Mutually exclusive stable motifs determine distinct attractors (distinct phenotypes). For example, in the model studied here the *openM1* stable motif and *closureM* conditionally stable motif are mutually exclusive (see Figure 3A). The stabilization of one of two mutually exclusive stable motifs at the expense of the other represents a bifurcation in the system’s trajectory toward a specific phenotype. The specific example in this work is the possibility of a trajectory toward closed stomata in the absence of ABA. Such a bifurcation can be viewed as a cellular decision point. Our work suggests a mechanism of cellular plasticity (phenotype switching): destabilization of the conditionally stable motif that underlies the phenotype by deactivating its condition. As seen in previous work (Deritei et al., 2019), stable motifs that are condition-free within the context of one model can become conditionally stable motifs in a broader model that encompasses the original model but includes more regulators and processes.

Our analysis of initial conditions of 17 nodes with uncertain states found that it is not possible to simultaneously have a baseline cumulative percentage of closure of 0 and also recapitulate the experimentally observed closure for constitutive activation of pH_*c*_, PA, NO or S1P (Jacob et al., 1999; Ng et al., 2001; Desikan et al., 2002; Coursol et al., 2003; Mishra et al., 2006; Gonugunta et al., 2008). This discrepancy suggests that the actual guard cell resting/open state does *not* correspond to the state farthest from the state associated with stomatal closure. In order to ensure optimal response over a range of conditions, certain nodes have to already be “primed” (be in their state associated with stomatal closure) prior to receiving the closure stimulus. The effectiveness of such priming has been documented in the case of Ca^2+^_*c*_ stimulus: when guard cells were pre-exposed to ABA or CO_2_, elevated Ca^2+^_*c*_ strongly activated S-type anion channels by shifting their Ca^2+^_*c*_ sensitivity to lower levels (Israelsson et al., 2006; Hubbard et al., 2012; Laanemets et al., 2013). This suggestion is also in accordance with previous experimental observations that the cellular changes underlying stomatal closure (e.g., induced by ABA) are not simply the reverse of the processes underlying stomatal opening (e.g., induced by light) (Assmann, 1993; Wang et al., 2001; Yin et al., 2013; He et al., 2018). Such “flexible” nodes may provide important portals for regulation by other stimuli to which multisensory guard cells also respond, including not only CO_2_ concentrations but also blue and red light, humidity, and pathogens (Sun et al., 2014; Murata et al., 2015; Assmann and Jegla, 2016; Engineer et al., 2016).

## Data Availability Statement

The original and updated network versions analyzed for this study can be found in the following github repository: https://github.com/parulm/Stomata_PP2Cs.

## Author Contributions

PM, SA, and RA designed the research and methodology, and wrote the manuscript. PM and RA performed the analyses. All authors contributed to the article and approved the submitted version.

## Conflict of Interest

The authors declare that the research was conducted in the absence of any commercial or financial relationships that could be construed as a potential conflict of interest.

## Supplementary Material

TABLE S1 The full names corresponding to the abbreviated node names in the 49-node ABA induced closure network.

TABLE S2 Initial conditions of all nodes in the model, reproduced from the 49-node model.

TABLE S3 The Boolean update function of each node in Model1.

TABLE S4 Attractors of Model1 and Model2.

TABLE S5 Attractors of Model1 with short-term memory.

TABLE S6 Simulation results from Model1 and Model2 with short-term memory for constitutive activation of nodes in the absence of ABA for initial conditions that ensure the absence of baseline closure.

TABLE S7 Simulation results for constitutive activation of each node in the absence of ABA in Model1 with short term memory for initial conditions that result in near zero baseline percentage of closure.

TEXT S1 Biological reasoning for the assumed slow decay of the activity of four network elements.

TEXT S2 Succession diagram for Model2.
