# Supplementary material for: A Guard Cell Abscisic Acid (ABA) Network Model That Captures the Stomatal Resting State
Source: Front Physiol. 2020 Aug 13;11:927. doi: 10.3389/fphys.2020.00927 (PMC7438572; doi:10.3389/fphys.2020.00927)
Supplement: TABLE S1 — The full names corresponding to the abbreviated node names in the 49-node ABA induced closure network. [file Table_1.docx]

Supplementary Information File for “A Guard Cell Abscisic Acid (ABA) Network Model That Captures the Stomatal Resting State**”**

**Table S1. The full names corresponding to the abbreviated node names in the 49-node ABA induced closure network.**

| **Node name in the network** | **Full name and description** |
| --- | --- |
| ABA | Abscisic acid |
| ABI1 | ABSCISIC ACID (ABA) INSENSITIVE 1 |
| ABI2 | ABSCISIC ACID (ABA) INSENSITIVE 2 |
| Actin Reorganization | Merged node of Actin Reorganization, Actin related protein complex (ARP Complex) and Stomatal Closure-related Actin Binding protein 1 (SCAB1). Both ARP Complex and SCAB1 are source nodes and assumed to be ON. The node also assumes the ON states of Phosphatidylinositol 3-phosphate (PtdInsP3) and Phosphatidylinositol 4-phosphate (PtdInsP4). |
| AnionEM | Anion efflux through the plasma membrane |
| AtRAC1 | Small GTPase RAC1 |
| Ca^2+^ ATPase | Ca^2+^ ATPases and Ca^2+/^H^+^ antiporters responsible for Ca^2+^ efflux from the cytosol |
| Ca^2+^_c_ | Cytosolic calcium |
| cADPR | Merged node of 8-nitro-cyclic guanosine monophosphate (8-nitro-cGMP), ADP (adenosine diphosphate)-ribosyl cyclase (ADPRc), Cyclic ADP-ribose (cADPR), and Nicotinamide adenine dinucleotide phosphate (NADPH). 8-nitro-cGMP is the only regulator of ADPRc, which is the only regulator of cADPR. NADPH is a source node assumed to be in the ON state, i.e. available in non-limiting concentrations. |
| CaIM | Merged node of Ca^2+^ influx across the plasma membrane, ABA Hypersensitive 1 (ABH1), Enhanced Response to Abscisic acid 1 (ERA1), Multidrug Resistance Protein 5 (MRP5), and Tobacco syntaxin-like SNARE (soluble N-ethylmaleimide-sensitive factor) attachment protein receptors (NtSyp121). ABH1, ERA1, MRP5, and NtSyp121 are source nodes and are fixed in the ON state. |
| cGMP | Merged node of Nitric Oxide-dependent Guanylate Cyclase 1 (NOGC1), Cyclic guanosine monophosphate (cGMP), and Guanosine 5'-triphosphate (GTP). NOGC1 is the only regulator of cGMP. GTP is a source node that is assumed to be in the ON state. |
| CIS | Ca^2+^ influx to the cytosol from intracellular stores |
| Closure | Stomatal closure |
| CPK3/21 | Calcium-dependent Protein Kinases 3 and 21 |
| CPK6/23 | Calcium-dependent Protein Kinases 6 and 23 |
| DAG | Merged node of Diacylglycerol and Phosphatidylinositol 4,5-bisphosphate (PtdIns(4,5)P2) and Phosphatidylinositol 4-phosphate (PtdInsP4). PtdInsP4 is a source node assumed to be ON and is the only regulator of PtdIns(4,5), which is also assumed to be ON as a result. |
| Depolarization | Plasma membrane depolarization |
| GHR1 | Guard cell Hydrogen peroxide Resistant 1 |
| H^+^ ATPase | H^+^ ATPase at the plasma membrane |
| H_2_O Efflux | Water efflux through the plasma membrane |
| HAB1 | Hypersensitive to ABA 1 |
| InsP3/6 | Merged node of Inositol-1,4,5 trisphosphate, Inositol hexakisphosphate and Phosphatidylinositol 4,5-bisphosphate (PtdIns(4,5)P2). PtdIns(4,5)P2 is regulated by a source node Phosphatidylinositol 4-phosphate (PtdInsP4) and both are assumed to be ON. |
| K^+^ Efflux | K^+^ efflux through the plasma membrane |
| KEV | K^+^ efflux from the vacuole to the cytosol |
| KOUT | K^+^ efflux through slowly activating outwardly-rectifying K^+^ channels through the plasma membrane |
| Malate | Malate |
| Microtubule Depolymerization | Microtubule depolymerization |
| MPK9/12 | Mitogen-activated Protein Kinases 9 and 12 |
| NIA1/2 | Nitrate reductase ½ |
| NO | Merged node of Nitric Oxide, Nitrite, and Nicotinamide adenine dinucleotide phosphate (NADPH). Nitrite and NADPH are source nodes assumed to be ON. |
| OST1 | Merged node of OPEN STOMATA 1 (protein kinase) and Plasma membrane intrinsic protein 2;1 (Aquaporin). OST1 is the only regulator of Aquaporin. |
| PA | Phosphatidic acid |
| PEPC | Phosphoenolpyruvate carboxylase |
| pH_c_ | Increase of the cytosolic pH level |
| PLC | Phospholipase C |
| PLDα | Phospholipase D α1 |
| PLDδ | Merged node of Phospholipase D δ and Glyceraldehyde-3-phosphate dehydrogenase subunits 1 and 2 (GAPC1/2). GAPC1/2 is a source node assumed to be ON. |
| PP2CA | Protein Phosphatase 2CA |
| QUAC1 | QuickLY-activating Anion Channel 1 |
| RCARs | Regulatory Components of ABA Receptor |
| ROP11 | Merged node of Small GTPase ROP11 and Guanine Exchange Factors 1, 4 and 10 (GEF1/4/10). GEF1/4/10 is a source node assumed to be OFF. |
| ROS | Merged node of Reactive oxygen species, NADPH oxidases AtRBOH D and F (RBOH), the protein phosphatase ROOTS CURL IN NAPHTHYLPHTHALAMIC ACID 1 (RCN1), Phosphatidylinositol 3-phosphate (PtdInsP3), and Nicotinamide adenine dinucleotide phosphate (NADPH). RBOH is the only regulator of ROS. RCN1, PtdInsP3, and NADPH are source nodes and are assumed to be ON. |
| S1P | Merged node of Sphingosine Kinases 1 and 2 (SPHK1/2), Sphingosine-1-phosphate (S1P), Heterotrimeric G protein α subunit 1 (GPA1), Sphingosine Phosphate Phosphatase 1 (SPP1), Sphingosine (Sph) and putative G protein–Coupled Receptor 1 (GCR1). SPHK1/2 is the only regulator of S1P, which is the only regulator of GPA1. SPP1 is a source node assumed to be OFF. Sph and GCR1 are source nodes assumed to be ON. |
| SLAC1 | Slow Anion Channel- associated 1 |
| SLAH3 | SLAC1 Homologue 3 |
| TCTP | Translationally controlled tumor protein |
| V-ATPase | Vacuolar proton ATPase |
| V-PPase | Merged node of Phosphatidylinositol 3-phosphate 5-kinase (PI3P5K), Phosphatidylinositol 3,5-bisphosphate (PtdIns(3,5)P2), and Vacuolar Proton Pyrophosphatase (V-PPase). PI3P5K is the only regulator of PtdIns(3,5)P2, which is the only regulator of V-PPase. |
| Vacuolar Acidification | Vacuolar acidification |

**Table S2. Initial conditions of all nodes in the model, reproduced from the 49-node model.**

Model1 and Model2 have the same initial conditions.

| **Initial State** | **Nodes** |
| --- | --- |
| OFF | Closure, H_2_O Efflux, Microtubule Depolymerization, Ca^2+^ ATPase, RCARs, pH_c_, Ca^2+^_c_, K^+^ Efflux, CIS, Vacuolar Acidification, PLDα, NIA1/2, CPK3/21, MPK9/12, NO, CaIM, PA, ROP11, Actin Reorganization, S1P, cGMP, ROS, InsP3/6, OST1 |
| ON | Malate, H^+^ ATPase, ABI1, ABI2, HAB1, PP2CA, CPK6/23 |
| Random | AnionEM, AtRAC1, Depolarization, GHR1, KEV, KOUT, PEPC, PLC, QUAC1, SLAC1, SLAH3, TCTP, V-ATPase, PLDδ, DAG, cADPR, V-PPase |

**Table S3. The Boolean update function of each node in Model1.**

The first column lists the target node, the second column lists its update function and the third column lists references that support the update function. These functions were first used in Maheshwari et al. (2019). * is used to denote the future state of a node.

| **Node** | **Regulatory function** | **References** |
| --- | --- | --- |
| ABI1 | ABI1* = not PA and (not RCARs or ROP11) and not ROS and pH_c_ | (Z. Li et al. 2012; Wenhua Zhang et al. 2004; Meinhard and Grill 2001; Leube, Grill, and Amrhein 1998) |
| ABI2 | ABI2* = (not RCARs or ROP11) and not ROS and not PA | (Ma et al. 2009; Meinhard, Rodriguez, and Grill 2002; Yu et al. 2012) |
| Actin Reorganization | Actin Reorganization* = not AtRAC1 | (Lemichez et al. 2001) |
| Anion EM | Anion EM* = SLAC1 or (QUAC1 and SLAH3) | (Imes et al. 2013; Hedrich 2012) |
| AtRAC1 | AtRAC1* = not ABA or ABI1 | (Lemichez et al. 2001) |
| CIS | CIS* = InsP3/6 or cADPR | (Guse 1999; Staxén et al. 1999; Lemtiri-Chlieh et al. 2003) |
| CPK3/21 | CPK3/21* = Ca^2+^_c_ or CPK3/21 | (Scherzer et al. 2012; Swatek et al. 2014) |
| Ca^2+^_c_ | Ca^2+^_c_* = (CaIM or CIS) and not Ca^2+^ ATPase | (S. Li, Assmann, and Albert 2006) |
| CaIM | CaIM* = Actin Reorganization or GHR1 | (Wei Zhang, Fan, and Wu 2007; Hua et al. 2012) |
| Ca^2+^ ATPase | Ca^2+^ ATPase* = Ca^2+^_c_ | (Sanders et al. 2002) |
| Closure | Closure* = Microtubule Depolymerization and H_2_O Efflux | (Jiang et al. 2014) |
| DAG | DAG* = PLC | (Munnik, Irvine, and Musgrave 1998) |
| Depolarization | Depolarization* = (AnionEM or Ca^2+^_c_ or KEV) and (not H^+^ ATPase or not K^+^ efflux) | (S. Li, Assmann, and Albert 2006) |
| GHR1 | GHR1* = not ABI2 and ROS | (Hua et al. 2012) |
| H_2_O Efflux | H_2_O Efflux* = AnionEM and OST1 and K^+^ efflux and not Malate | (Grondin et al. 2015) |
| HAB1 | HAB1* = not RCARs and not ROS | (Sridharamurthy et al. 2014) |
| H^+^ ATPase | H^+^ ATPase* = not pH_c_ and not Ca^2+^_c_ and not ROS | (X. Zhang et al. 2004; Kinoshita, Nishimura, and Shimazaki 1995; Luo, Morsomme, and Boutry 1999) |
| InsP3/6 | InsP3/6* = PLC | (Otterhag, Sommarin, and Pical 2001; Boss and Im 2012) |
| KEV | KEV* = Vacuolar Acidification or Ca^2+^_c_ | (Ward and Schroeder 1994) |
| KOUT | KOUT* = (not NO or not ROS or pH_c_) and Depolarization | (Miedema and Assmann 1996; Köhler, Hills, and Blatt 2003; Sokolovski and Blatt 2004) |
| K^+^ Efflux | K^+^ Efflux* = KEV and KOUT | (Ward and Schroeder 1994; Hosy et al. 2003) |
| MPK9/12 | MPK9/12* = Ca^2+^_c_ or MPK9/12 | (Boss and Im 2012; Jiang et al. 2014) |
| Malate | Malate* = PEPC and not ABA and not AnionEM | (S. Li, Assmann, and Albert 2006; Du, Aghoram, and Outlaw Jr 1997; Dittrich and Raschke 1977) |
| Microtubule Depolymerization | Microtubule Depolymerization* = TCTP or Microtubule Depolymerization | (Kim et al. 2012; Gardner, Zanic, and Howard 2013) |
| NIA1/2 | NIA1/2* = ROS | (Bright et al. 2006) |
| NO | NO* = NIA1/2 | (Desikan et al. 2002) |
| cGMP | cGMP* = NO | (Mulaudzi et al. 2011) |
| OST1 | OST1* = (not ABI1 and not HAB1) or (not PP2CA and not ABI2) or (not ABI1 and not ABI2) or (not HAB1 and not PP2CA) or (not HAB1 and not ABI2) or (not ABI1 and not PP2CA) | (Park et al. 2009; Umezawa et al. 2009; Nishimura et al. 2010; Merlot et al. 2001; Saez et al. 2006; Rubio et al. 2009) |
| PA | PA* = PLDδ or PLDα or DAG | (Munnik, Irvine, and Musgrave 1998; Distefano et al. 2008) |
| PEPC | PEPC* = not ABA | (Du, Aghoram, and Outlaw Jr 1997) |
| V-PPase | V-PPase* = ABA | (Bak et al. 2013) |
| PLC | PLC* = Ca^2+^_c_ | (Otterhag, Sommarin, and Pical 2001) |
| PLDα | PLDα* = S1P and Ca^2+^_c_ | (L. Guo and Wang 2012; Pappan and Wang 2013) |
| PLDδ | PLDδ* = NO or ROS | (Distéfano et al. 2012; Liang Guo et al. 2012) |
| PP2CA | PP2CA* = not RCARs and not ROS | (Meinhard and Grill 2001; Meinhard, Rodriguez, and Grill 2002; Sridharamurthy et al. 2014; Antoni et al. 2012) |
| QUAC1 | QUAC1* = OST1 and Ca^2+^_c_ | (Imes et al. 2013; Sasaki et al. 2010) |
| ROS | ROS* = pH_c_ and not ABI1 and OST1 and S1P and PA | (Acharya et al. 2013; Y. Zhang et al. 2009; Murata et al. 2001; Wei Zhang, Jeon, and Assmann 2011; Suhita et al. 2004; Coursol et al. 2003; Kwak et al. 2002) |
| RCARs | RCARs* = ABA | (Ma et al. 2009; Park et al. 2009) |
| SLAC1 | SLAC1* = (CPK3/21 or CPK6/23) and MPK9/12 and OST1 and GHR1 and not ABI1 and not PP2CA and not ABI2 and pH_c_ | (Acharya et al. 2013; Scherzer et al. 2012; Hua et al. 2012; Geiger et al. 2009; Wang et al. 2001) |
| SLAH3 | SLAH3* = CPK6/23 and CPK3/21 and not ABI1 | (Geiger et al. 2011; Scherzer et al. 2012) |
| S1P | S1P* = PA or ABA | (Coursol et al. 2003; Liang Guo et al. 2011) |
| TCTP | TCTP* = Ca^2+^_c_ | (Kim et al. 2012) |
| V-ATPase | V-ATPase* = Ca^2+^_c_ | (Tang et al. 2012) |
| Vacuolar Acidification | Vacuolar Acidification* = V-PPase or Vacuolar Acidification | (Martinoia, Maeshima, and Neuhaus 2007) |
| cADPR | cADPR* = cGMP and ROS and NO | (Joudoi et al. 2013) |
| pH_c_ | pH_c_* = ((OST1 and not ABI2 and not ABI1) or Ca^2+^_c_) and Vacuolar Acidification | (Bak et al. 2013; Islam et al. 2010; Gonugunta et al. 2008) |

**Table S4. Attractors of Model1 and Model2.**

Model1 and Model2 have identical attractors. Nineteen attractors are reachable when 17 nodes are initialized randomly (due to lack of information on their resting states). The attractors are summarized in five different sections of the table. The first section indicates the only attractor possible in the presence of ABA; this attractor corresponds to closed stomata. The second section summarizes the sole attractor possible that corresponds to closed stomata in the absence of ABA; this attractor is denoted A0 in Figure 3 and differs from the attractor reached in the presence of ABA in the state of the five nodes indicated in blue font. This attractor A0 is reached by the constitutive activation of the nodes listed in the last row of Table S5; this is also the attractor that is reached when simulating the presence of external calcium as the fixed ON state of the node CaIM. The third section contains the attractor reached by simulating external calcium as the fixed ON state of the node Ca^2+^_c_. This attractor differs from the one detailed in the second section of the table in that the nodes that oscillated there have a sustained ON state here. The fourth section indicates the common node states of the 16 attractors that correspond to open stomata in the absence of ABA. The fifth section details the differences in these 16 attractors. In this section the nodes marked by X, namely, K^+^ Efflux, Depolarization and KOUT, oscillate between OFF and ON. These oscillations are conditional to Vacuolar Acidification stabilizing in the ON state and arise from a negative feedback loop formed by the three nodes. Each of these have an average ON/OFF period of 1.77 time steps. This average ON/OFF period can be calculated by the method shown in Maheshwari et al. (2019). The nodes in orange font stabilize in a state opposite to their initial state (corresponding to open stomata) and the nodes in light green font stabilize in their initial state. In the second and third section of this table, blue font indicates the nodes that differ in their state from the closed stomata attractor induced by ABA (shown in the first section of the table).

| **1. Attractor associated with closure in the presence of ABA** | | | **Node count** | | **Nodes** | | | | | | |
| --- | --- | --- | --- | --- | --- | --- | --- | --- | --- | --- | --- |
| Stabilized in the ON state | | | 30 | | Actin reorganization, AnionEM, cADPR, CaIM, CIS, Closure, cGMP, CPK3/21, CPK6/23, Depolarization, GHR1, H_2_O Efflux, K^+^ Efflux, KEV, KOUT, MPK 9/12, Microtubule Depolymerization, NIA1/2, NO, OST1, pH_c_, V-PPase, PA, PLDδ, RCARs, ROS, S1P, SLAC1, SLAH3, Vacuolar Acidification | | | | | | |
| Stabilized in the OFF state | | | 9 | | ABI1, ABI2, AtRAC1, HAB1, H^+^ ATPase, Malate, PEPC, PP2CA, ROP11 | | | | | | |
| Oscillates with average ON/OFF period of 1.33 time steps | | | 2 | | Ca^2+^_c_, Ca^2+^ ATPase | | | | | | |
| Oscillates with average ON/OFF period of 1.77 time steps | | | 5 | | PLC, PLDα, QUAC1, TCTP, V-ATPase | | | | | | |
| Oscillates with average ON/OFF period of 2.2 time steps | | | 2 | | DAG, InsP3/6 | | | | | | |
| **2. Attractor associated with closure in the absence of ABA** | | | **Node count** | | **Nodes** | | | | | | |
| Stabilized in the ON state | | | 29 | | AnionEM, AtRAC1, cADPR, CaIM, CIS, Closure, cGMP, CPK3/21, CPK6/23, Depolarization, GHR1, H_2_O Efflux, K^+^ Efflux, KEV, KOUT, MPK 9/12, Microtubule Depolymerization, NIA1/2, NO, OST1, pH_c_, PA, PEPC, PLDδ, ROS, S1P, SLAC1, SLAH3, Vacuolar Acidification | | | | | | |
| Stabilized in the OFF state | | | 10 | | Actin reorganization, ABI1, ABI2, HAB1, H^+^ ATPase, Malate, PP2CA, RCARs, ROP11, V-PPase | | | | | | |
| Oscillates with average ON/OFF period of 1.33 time steps | | | 2 | | Ca^2+^_c_, Ca^2+^ ATPase | | | | | | |
| Oscillates with average ON/OFF period of 1.77 time steps | | | 5 | | PLC, PLDα, QUAC1, TCTP, V-ATPase | | | | | | |
| Oscillates with average ON/OFF period of 2.2 time steps | | | 2 | | DAG, InsP3/6 | | | | | | |
| **3. Attractor associated with closure in the case of elevated Ca^2+^_c_** | | | **Node count** | | **Nodes** | | | | | | |
| Stabilized in the ON state | | | 38 | | AnionEM, AtRAC1, cADPR, Ca^2+^_c_, Ca^2+^ATPase, CaIM, CIS, Closure, cGMP, CPK3/21, CPK6/23, DAG, Depolarization, GHR1, H_2_O Efflux, InsP3/6, K^+^ Efflux, KEV, KOUT, MPK 9/12, Microtubule Depolymerization, NIA1/2, NO, OST1, pH_c_, PA, PEPC, PLC, PLDα, PLDδ, ROS, S1P, SLAC1, SLAH3, Vacuolar Acidification, , QUAC1, TCTP, V-ATPase, | | | | | | |
| Stabilized in the OFF state | | | 10 | | Actin reorganization, ABI1, ABI2, HAB1, H^+^ ATPase, Malate, PP2CA, RCARs, ROP11, V-PPase | | | | | | |
| **4. Attractors associated to lack of closure in the absence of ABA** | | | **Node count** | | **Nodes** | | | | | | |
| Stabilized in the ON state | | | 8 | | ABI2, AtRAC1, CPK6/23, H^+^ ATPase, HAB1, Malate, PEPC, PP2CA | | | | | | |
| Stabilized in the OFF state | | | 31 | | ABI1, Actin Reorganization, AnionEM, Ca^2+^, cADPR, CaIM, Ca^2+^ ATPase, cGMP, CIS, Closure, DAG, GHR1, H_2_O Efflux, InsP3/6, NIA1/2, NO, OST1, QUAC1, ROS, pH_c_, PA, V-PPase, PLC, PLDα, PLDδ, RCARs, ROP11, SLAC1, S1P, TCTP, V-ATPase | | | | | | |
| Stabilized in either the ON or OFF state | | | 6 | | CPK3/21, MPK9/12, KEV, Microtubule Depolymerization, SLAH3, Vacuolar Acidification | | | | | | |
| Stabilized in the OFF state or oscillates with average ON/OFF period of 1.77 time steps | | | 3 | | Depolarization, KOUT, K^+^ Efflux | | | | | | |
|  | | |  | |  | | | | | | |
| **5. List of the nodes whose states are different in the attractors corresponding to open stomata in the absence of ABA** | | | | | | | | | | | |
| Attractor | Microtubule Depolymerization | Vacuolar Acidification | | Depolarization | | K^+^ Efflux | SLAH3 | CPK3/21 | KOUT | KEV | MPK9/12 |
| A1 | 1 | 1 | | X | | X | 1 | 1 | X | 1 | 1 |
| A2 | 1 | 0 | | 0 | | 0 | 1 | 1 | 0 | 0 | 1 |
| A3 | 0 | 1 | | X | | X | 1 | 1 | X | 1 | 1 |
| A4 | 0 | 0 | | 0 | | 0 | 1 | 1 | 0 | 0 | 1 |
| A5 | 1 | 1 | | X | | X | 1 | 1 | X | 1 | 0 |
| A6 | 1 | 0 | | 0 | | 0 | 1 | 1 | 0 | 0 | 0 |
| A7 | 0 | 1 | | X | | X | 1 | 1 | X | 1 | 0 |
| A8 | 0 | 0 | | 0 | | 0 | 1 | 1 | 0 | 0 | 0 |
| A9 | 1 | 1 | | X | | X | 0 | 0 | X | 1 | 1 |
| A10 | 1 | 0 | | 0 | | 0 | 0 | 0 | 0 | 0 | 1 |
| A11 | 0 | 1 | | X | | X | 0 | 0 | X | 1 | 1 |
| A12 | 0 | 0 | | 0 | | 0 | 0 | 0 | 0 | 0 | 1 |
| A13 | 1 | 1 | | X | | X | 0 | 0 | X | 1 | 0 |
| A14 | 1 | 0 | | 0 | | 0 | 0 | 0 | 0 | 0 | 0 |
| A15 | 0 | 1 | | X | | X | 0 | 0 | X | 1 | 0 |
| A16 | 0 | 0 | | 0 | | 0 | 0 | 0 | 0 | 0 | 0 |

**Table S5. Attractors of Model1 with short-term memory.**

The model can lead to different attractors in the presence of ABA depending on the duration of the memory on the nodes CPK3/21, MPK9/12, Microtubule Depolymerization, and Vacuolar Acidification. When the memory duration exceeds 2 time-steps for Vacuolar Acidification, 3 time-steps for CPK3/21 and MPK9/12, 5 time-steps for Microtubule Depolymerization, then there is only one attractor that corresponds to closed stomata. This attractor is the same as the closed stomata attractor obtained by the sustained presence of ABA, indicated in the first part of Table S4. This attractor is repeated here as the first part of this table. When the memory duration is insufficient, there is a single attractor in which some nodes are fixed, and others oscillate. Closure is also one of the nodes that oscillates. The oscillation period of these oscillating nodes depends on the memory duration. Here we report the oscillation periods of the nodes when the memory duration is 1 (i.e., the smallest possible). This attractor is summarized in the second part of this table. Model1 with short-term memory has only one open stomata attractor in the absence of any signal. This attractor is detailed in the third part of this table and is the same as attractor A16 in Table S4. The nodes in orange font stabilize in a state opposite to their initial state (corresponding to open stomata) and the nodes in light green font stabilize in their initial state.

| **Attractor associated to closure in the presence of ABA for large memory duration** | **Node count** | **Nodes** |
| --- | --- | --- |
| Stabilized in the ON state | 30 | Actin reorganization, AnionEM, cADPR, CaIM, CIS, Closure, cGMP, CPK3/21, CPK6/23, Depolarization, GHR1, H_2_O Efflux, K^+^ Efflux, KEV, KOUT, MPK 9/12, Microtubule Depolymerization, NIA1/2, NO, OST1, pH_c_, V-PPase, PA, PLDδ, RCARs, ROS, S1P, SLAC1, SLAH3, Vacuolar Acidification |
| Stabilized in the OFF state | 9 | ABI1, ABI2, AtRAC1, HAB1, H^+^ ATPase, Malate, PEPC, PP2CA, ROP11 |
| Oscillates with average ON/OFF period of 1.33 time steps | 2 | Ca^2+^_c_, Ca^2+^ ATPase |
| Oscillates with average ON/OFF period of 1.77 time steps | 5 | PLC, PLDα, QUAC1, TCTP, V-ATPase |
| Oscillates with average ON/OFF period of 2.2 time steps | 2 | DAG, InsP3/6 |
| **Attractor associated to oscillating closure in the presence of ABA for small memory duration** | **Node count** | **Nodes** |
| Stabilized in the ON state | 22 | Actin Reorganization, cADPR, CaIM, CIS, cGMP, CPK6/23, Depolarization, GHR1, K^+^ Efflux, KEV, KOUT, NIA1/2, NO, OST1, pH_c_, PA, PLDδ, RCARs, ROS, S1P, V-PPase, Vacuolar Acidification |
| Stabilized in the OFF state | 9 | ABI1, ABI2, AtRAC1, H^+^ ATPase, HAB1, Malate, PEPC, PP2CA, ROP11 |
| Oscillates with average ON/OFF period of 1.33 time steps | 2 | Ca^2+^ ATPase, Ca^2+^_c_ |
| Oscillates with average ON/OFF period of 1.77 time steps | 5 | PLDα, PLC, TCTP, QUAC1, V-ATPase |
| Oscillates with average ON/OFF period of 2 time steps | 2 | InsP3/6, DAG |
| Oscillates with average ON/OFF period of 2.6 time steps | 1 | Closure |
| Oscillates with unequal average ON/OFF periods; ON, OFF periods in brackets | 7 | AnionEM (4.3, 1.5), CPK3/21 (2.5, 1.2), H_2_O Efflux (4.7, 1.6), MPK9/12 (2.5, 1.2), Microtubule Depolymerization (2.9, 1.6), SLAC1 (3.1, 1.7), SLAH3 (3.0, 1.6) |
| **Attractor associated to lack of closure in the absence of ABA** | **Node count** | **Nodes** |
| Stabilized in the ON state | 8 | ABI2, AtRAC1, CPK6/23, H^+^ ATPase, HAB1, Malate, PEPC, PP2CA |
| Stabilized in the OFF state | 40 | ABI1, Actin Reorganization, AnionEM, Ca^2+^, cADPR, CaIM, Ca^2+^ ATPase, cGMP, CIS, Closure, CPK3/21, DAG, Depolarization, GHR1, H_2_O Efflux, InsP3/6, KEV, KOUT, K^+^ Efflux, Microtubule Depolymerization, MPK9/12, NIA1/2, NO, OST1, QUAC1, ROS, pH_c_, PA, V-PPase, PLC, PLDδ, PLDδ, RCARs, ROP11, SLAC1, SLAH3, S1P, TCTP, V-ATPase, Vacuolar Acidification |

**Table S6. Simulation results from Model1 and Model2 with short-term memory for constitutive activation of each node in the absence of ABA for initial conditions that ensure the absence of baseline closure.**

We summarize in this table the simulation results of constitutive activation (CA) of each node in the network. We performed 500 simulations over 50 time-steps in each setting. For each of the 4 cases summarized in this table the cumulative percentage of closure (CPC) of the WT simulation (where no node was constitutively activated) was 0.0. The first column lists the various response categories (see Methods). The second to fifth columns list the cases of node CA that belong to this category, indicating the CPC values in parentheses. We studied two model versions, namely when Ca^2+^_c_ inhibits ABI2 via PA (Model1) and when Ca^2+^_c_ inhibits ABI2 directly (Model2). For each model version, we considered two initial conditions: the initial condition furthest from closure, where the 17 nodes with unknown initial (pre-stimulus) state are initialized in the opposite of the state they achieve in the closure attractor, and the least restricted initial condition that gives zero baseline closure percentage and CPC. Node perturbations marked in blue agree with experiments and those shown in red disagree. No experimental data were reported in the literature for the remaining nodes. When using the furthest from closure initial condition in Model1 there are 13 cases of agreement with experiments and 4 cases of disagreement. These 4 cases are explained in detail in the main text. When using the least restricted initial condition (where cADPR=OFF, GHR1=OFF, AtRAC1=ON, PLC=OFF, PLDδ=OFF, and DAG=OFF is imposed while 11 nodes are initialized randomly), there are 14 cases of agreement and 3 cases of disagreement. The difference between the results is in the constitutive activation of PLDα, PA and NO. When using the furthest from closure initial condition in Model2, there are 13 cases of agreement with experiments and 4 cases of disagreement; these cases are the same as those found for Model1. The same response categories are obtained in Model2 when using the least restricted initial condition that ensures the absence of a baseline closure percentage (i.e. when only restricting cADPR=OFF, GHR1=OFF, AtRAC1=ON and PLC=OFF and initializing 13 nodes randomly).

| Model version | Model1 | | Model2 | |
| --- | --- | --- | --- | --- |
| Response category | Furthest from closure initial condition | Least restricted initial condition | Furthest from closure initial condition | Least restricted initial condition |
| Close to baseline | SLAH3 (0.0), PLDα (0.0), Microtubule Depolymerization (0.0), Ca^2+^ ATPase (0.0), NIA1/2 (0.0), NO (0.0), PA (0.0), CPK3/21 (0.0), KOUT (0.0), CPK6/23 (0.0), V-PPase (0.0), V-ATPase (0.0), S1P (0.0), H^+^ ATPase (0.0), PEPC (0.0), H_2_O Efflux (0.0), ABI1 (0.0), SLAC1 (0.0), OST1 (0.0), DAG (0.0), TCTP (0.0), ROP11 (0.0), MPK9/12 (0.0), pH_c_ (0.0), K^+^ efflux (0.0), Vacuolar Acidification (0.0), PP2CA (0.0), Depolarization (0.0), cGMP (0.0), RCARs (0.0), QUAC1 (0.0), KEV (0.0), Malate (0.0), ABI2 (0.0), AtRAC1 (0.0), HAB1 (0.0), AnionEM (0.0), PLDδ (0.0) | SLAH3 (0.0), Microtubule Depolymerization (0.0), Ca^2+^ ATPase (0.0), CPK3/21 (0.0), KOUT (0.0), CPK6/23 (0.0), V-PPase (0.0), V-ATPase (0.0), S1P (0.0), H^+^ ATPase (0.0), PEPC (0.0), ABI1 (0.0), SLAC1 (0.0), TCTP (0.0), ROP11 (0.0), MPK9/12 (0.0), pH_c_ (0.0), K^+^ efflux (0.0), Vacuolar Acidification (0.0), PP2CA (0.0), Depolarization (0.0), cGMP (0.0), QUAC1 (0.0), KEV (0.0), Malate (0.0), ABI2 (0.0), AtRAC1 (0.0), HAB1 (0.0), AnionEM (0.0), OST1 (0.02), RCARs (0.03), NIA1/2 (1.05) | SLAH3 (0.0), PLDα (0.0), Microtubule Depolymerization (0.0), Ca^2+^ ATPase (0.0), NIA1/2 (0.0), NO (0.0), PA (0.0), CPK3/21 (0.0), KOUT (0.0), CPK6/23 (0.0), V-PPase (0.0), V-ATPase (0.0), S1P (0.0), H^+^ ATPase (0.0), PEPC (0.0), H_2_O Efflux (0.0), ABI1 (0.0), SLAC1 (0.0), OST1 (0.0), DAG (0.0), TCTP (0.0), ROP11 (0.0), MPK9/12 (0.0), pH_c_ (0.0), K^+^ efflux (0.0), Vacuolar Acidification (0.0), PP2CA (0.0), Depolarization (0.0), cGMP (0.0), RCARs (0.0), QUAC1 (0.0), KEV (0.0), Malate (0.0), ABI2 (0.0), AtRAC1 (0.0), HAB1 (0.0), AnionEM (0.0), PLDδ (0.0) | SLAH3 (0.0), PLDα (0.0), Microtubule Depolymerization (0.0), Ca^2+^ ATPase (0.0), NIA1/2 (0.0), NO (0.0), PA (0.0), CPK3/21 (0.0), KOUT (0.0), CPK6/23 (0.0), V-PPase (0.0), V-ATPase (0.0), S1P (0.0), H^+^ ATPase (0.0), PEPC (0.0), ABI1 (0.0), SLAC1 (0.0), DAG (0.0), TCTP (0.0), ROP11 (0.0), MPK9/12 (0.0), pH_c_ (0.0), K^+^ efflux (0.0), Vacuolar Acidification (0.0), PP2CA (0.0), Depolarization (0.0), cGMP (0.0), QUAC1 (0.0), KEV (0.0), Malate (0.0), ABI2 (0.0), AtRAC1 (0.0), HAB1 (0.0), AnionEM (0.0), PLDδ (0.0), OST1 (0.02), RCARs (0.2) |
| Slightly increased | - | H_2_O Efflux (2.57), NO (3.06), DAG (4.31), PLDδ (4.57), PLDα (4.7), PA (6.93) | - | H_2_O Efflux (2.55) |
| Significantly increased (100% closure) | CIS (25.47), GHR1 (26.6), cADPR (26.79), Actin Reorganization (27.05), InsP3/6 (27.81), CaIM (29.06), PLC (32.3), ROS (33.29), Ca^2+^_c_ (44.8) | cADPR (27.01), InsP3/6 (27.33), CIS (27.36), Actin Reorganization (27.48), CaIM (27.59), GHR1 (28.32), PLC (33.11), ROS (34.3), Ca^2+^_c_ (44.67) | CIS (21.01), cADPR (21.37), GHR1 (21.98), Actin Reorganization (23.86), CaIM (24.2), InsP3/6 (24.66), PLC (27.98), ROS (33.11), Ca^2+^_c_ (45.9) | Actin Reorganization (22.44), InsP3/6 (22.71), CIS (22.96), cADPR (23.3), CaIM (23.32), GHR1 (23.45), PLC (27.49), ROS (34.14), Ca^2+^_c_ (45.97) |

**Table S7.** **Simulation results for constitutive activation of each node in the absence of ABA in Model1 with short term memory for initial conditions that result in near zero baseline percentage of closure.**

In section 3.4 of the main text, we present the least restricted initial condition that ensures that the transient baseline percentage of closure is zero and in Table S6 we present the simulation results for constitutive activation of each node using this initial condition and also the initial condition furthest from closure. We also tried less restricted initial conditions, where only a subset of cADPR=OFF, GHR1=OFF, AtRAC1=ON, PLC=OFF, PLDδ=OFF, and DAG=OFF is imposed, and identified the effect on the baseline closure, and on the response to node constitutive activations for which experimental results are available. We find and list here three cases in Model1 which have near zero baseline cumulative percentage of closure (CPC); we find no such cases in Model2. The first row gives the initial condition. For each of the second, third and fourth columns, the first row lists the nodes that are initialized in a state opposite to their corresponding state in closed stomata attractor; the rest of the 17 nodes with unknown pre-stimulus state are initialized with a random state. In the following rows, we summarize the 17 cases of constitutive activation (CA) of nodes for which experimental evidence exists along with the CPC values for each of them. We performed 500 simulations over 50 time-steps in each setting. The first column lists the various response categories (see Methods). The second through fourth columns list the cases of node CA that belong to this category in increasing order of CPC values. Node perturbations marked in bold agree with experiments, and those shown in normal font disagree. In each of these cases, there are 15 cases of agreement with experiments and 2 cases of disagreement. However, the discrepancy in CPC values changes for different initial conditions. The last two rows give the baseline CPC and the peak transient percentage of closure respectively for each of these cases.

| Response category | GHR1, cADPR, PLC, AtRAC1 | GHR1, cADPR, PLC, AtRAC1, DAG | GHR1, cADPR, PLC, AtRAC1, PLDδ |
| --- | --- | --- | --- |
| Close to baseline | **ABI1**(0.0), **ABI2**(0.0), **H^+^ ATPase**(0.0), **TCTP**(0.21)**, ROP11**(0.03)**, Microtubule Depolymerization**(0.24), S1P(0.02), **AtRAC1**(0.23), **PP2CA**(0.03) | **ABI1**(0.0), **ABI2**(0.0), **H^+^ ATPase**(0.0), **TCTP**(0.14)**, ROP11**(0.05)**, Microtubule Depolymerization**(0.14), S1P(0.20), **AtRAC1**(0.05), **PP2CA**(0.06) | **ABI1**(0.0)**, ABI2**(0.0), **H^+^ ATPase**(0.02)**, TCTP**(0.05)**, ROP11**(0.18)**, Microtubule Depolymerization**(0.07), S1P(0.06)**, AtRAC1**(0.08)**, PP2CA**(0.10) |
| Slightly increased | **pH_c_**(2.03), **NO**(5.31), PLDα(5.72), **PA**(7.29) | **pH_c_**(1.19), **NO**(3.84), PLDα(3.84), **PA**(7.03) | **pH_c_**(0.92), **NO**(4.45), PLDα(5.17), **PA**(6.69) |
| Significantly increased (100% closure) | **cADPR**(26.96), **InsP3/6**(28.53)**, ROS**(33.98)**, CaIM**(28.44) | **cADPR**(27.63), **InsP3/6**(27.74)**,**  **ROS**(34.26)**, CaIM**(27.85) | **cADPR**(27.55), **InsP3/6**(27.47)**, ROS**(34.03)**, CaIM**(28.25) |
| Baseline CPC | 0.18 | 0.06 | 0.11 |
| Peak transient % of closure | 0.8% | 0.4% | 0.2% |

**Text S1. Biological reasoning for the assumed slow decay of the activity of four network elements.**

The precursor model published in Albert et al. (2017) introduces the assumption of self-sustaining activity of each of CPK3/21, MPK9/12, Vacuolar Acidification, and Microtubule Depolymerization (see Section 2.2.2). Here, we reproduce from Albert et al. (2017) the sentences that indicate the biological basis for these assumptions.

CPK3/21* = Ca^2+^_c_ or CPK3/21
“Cytosolic calcium activates CPK3 and CPK21 by binding to their EF hand calcium binding motifs. We assume that once activated by Ca^2+^_c_, CPK3 and CPK21 remain in the active state through autophosphorylation (Swatek et al. 2014), and implement this assumption by using the “or” operator.”

MPK9/12* = Ca^2+^_c_ or MPK9/12
“We assume that once activated, MPK9 and MPK12 remain active due to autophosphorylation (Nagy et al. 2015), and implement this assumption by using the “or” operator.”

Microtubule depolymerization* = TCTP or Microtubule depolymerization
“Microtubule depolymerization continues forward once initiated by a process termed catastrophe (Gardner, Zanic, and Howard 2013); we assume that it is not reversed during the ABA signaling process and implement this assumption by using the “or” operator.”

Vacuolar acidification* = V-ATPase or V-PPase or Vacuolar acidification
“We assume that the vacuolar acidification state is sustained for a longer period, and implement this assumption as a positive self-regulation. This assumption is necessary in order to allow the possibility of closure in response to internal closure signals, e.g. supply of S1P or Ca^2+^ (Schwartz 1985).”

These descriptions indicate that for three of the four nodes, there is a molecular mechanism that slows down the decay of the node’s activity. In other words, the information in the literature supports the assumption of short-term memory in our model (e.g. the maintenance of the activity of CPK3/21 for a certain duration after the cytosolic Ca^2+^ level is no longer high). Here we describe possible experimental methods to determine the duration of this memory. The resulting information could guide future research and result in an even more precise Boolean model. To test the duration of the memory of CPK3/21, one would need to measure phospho-CPK3 and phospho-CPK21 in a time sequence where Ca^2+^ is provided for a sufficient time to establish the phosphorylation of CPK3 and CPK21, then taken away. A similar experiment performed for MPK9 and MPK12 would test the maintenance of MPK9/12 activation. While such experiments have not been reported, and would be difficult to perform, phosphorylation as an auto-activation mechanism is well-known to be reversed by phosphatases. Thus, it is possible that the process of ABA-induced stomatal closure also includes the activation of a phosphatase that reverses the auto-phosphorylation of CPKs and MPKs. An experiment measuring the time period of microtubule repolymerization following catastrophe would evaluate the persistence of microtubule depolymerization. Here also, it is known that the memory is finite since microtubule depolymerization as a permanent effect would lead to loss of the cellular cytoskeleton. To test the memory of vacuolar acidification, the pH level of the vacuole would need to be measured in a time course where the activity of the vacuolar ATPase or the vacuolar PPase is induced and then stopped. This would also be a difficult experiment. In summary, there is ample conceptual support for both the existence, and the finite duration, of a memory effect in the link between cytosolic Ca^2+^ and these four nodes.

**Text S2. Succession diagram for Model2.**

Model2 assumes that Ca^2+^_c_ directly inhibits ABI2 by an edge, as described in Maheshwari et al. (2019). We first consider the model with persistent activity of the nodes CPK3/21, MPK9/12, Vacuolar Acidification, and Microtubule Depolymerization. This model has the same stable motifs in the presence of ABA as Model1. In the absence of ABA, the stable motifs *openM1* and *openM2* and the two conditional oscillatory motifs are preserved. This model has two additional stable motifs, shown in Figure S1, that significantly overlap the *openM1* and *openM2* motifs. We refer to these motifs as *openM3* and *openM4*. The *closureM* conditionally stable motif is slightly different in Model2 compared to Model1. Specifically, the edge from PA to ABI2 is missing in the *closureM* motif of Model2. The absence of this edge prevents PA from being an internal driver of the *closureM* motif. Nevertheless, the fixed ON state or sustained oscillations of Ca^2+^_c_ are still external drivers of the *closureM* motif.


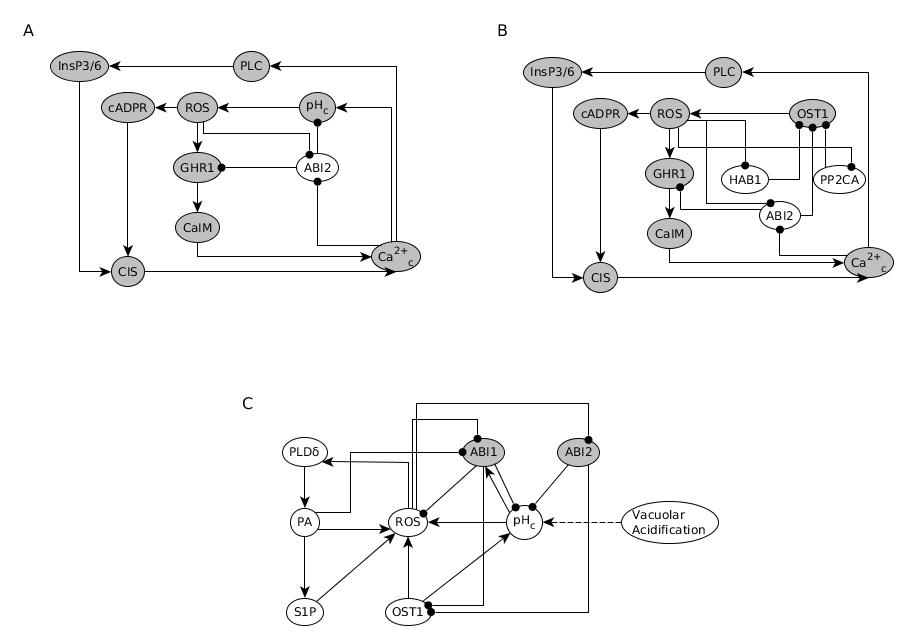


**Figure S1. Motifs in Model2 in the absence of ABA that are different from the motifs of Model1.** The network of Model2 has all the motifs of Model1, shown in Figure 3, and it also has two new motifs *openM3* (panel A) and *openM4* (panel B*)*. The *closureM* motif (panel C) is slightly different in Model2 since it does not have the edge from PA to ABI2.

Model2 has an identical motif succession diagram in the presence of ABA as Model1 (Figure 4). The motif succession diagram in the absence of ABA includes the succession diagram of Model1 (shown in Figure 3). It additionally includes branches that contain the *openM3* and *openM4* motifs and end in an attractor corresponding to open stomata. Examples of succession diagram branches containing these motifs are shown in Figure S2.


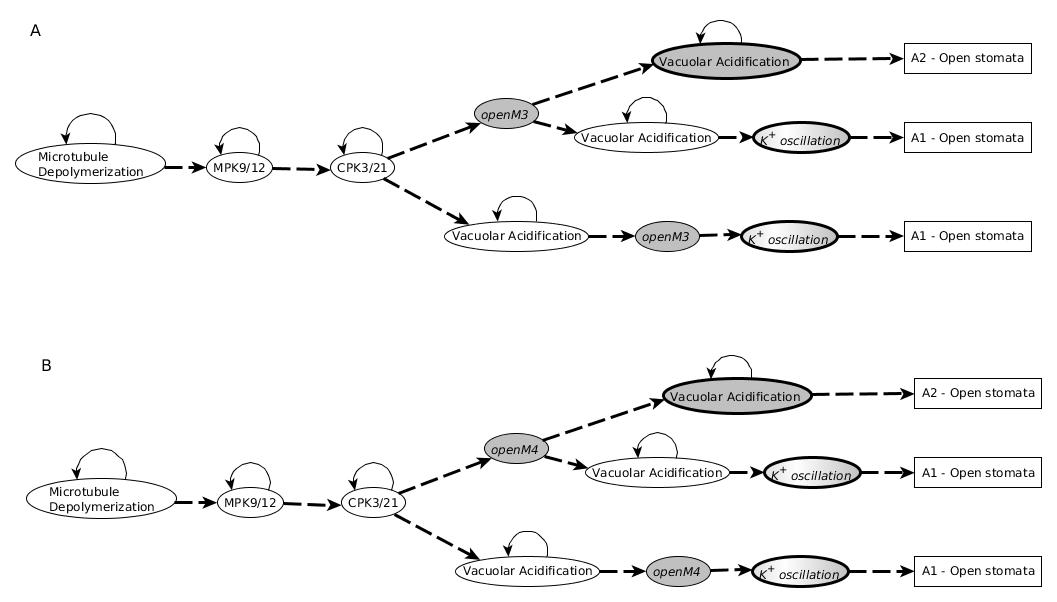


**Figure S2. Succession diagram branches containing motifs *openM3* and *openM4*.** Both of these motifs induce a very similar behavior as the motif *openM1* shown in Figure 3 – they correspond to open stomata attractors and their stabilization is a condition for the conditionally stable motifs Microtubule Depolymerization = 0, CPK3/21 = 0, Vacuolar Acidification = 0, and MPK9/12 = 0. The trajectories involving either these motifs have the possibility of establishing the *K^+^ oscillation* conditional oscillatory motif if Vacuolar Acidification = 1 has been established. Panels A and B show the succession diagram when the first motif to stabilize is Microtubule Depolymerization = 1, the second motif to stabilize is MPK9/12 = 1 and the third motif to stabilize is CPK3/21 = 1 for the trajectories that contain the stabilization of *openM3* and *openM4* respectively. The attractors resulting from these trajectories are the same as those described in Table S4.

The new motifs and the extended succession diagram of Model2 does not cause a difference between the two models in terms of biological results. As described in Maheshwari et al. (2019), the attractors of the two models are the same. The percentage of closure of Model1 in the absence of ABA is 30.28%, while the percentage of closure of Model2 is 21.3%. The smaller probability of reaching the closure attractor in the absence of ABA in Model2 is consistent with the higher number of stable motif successions leading to an open stomata attractor in Model2. It is also consistent with the slightly weaker inhibition of ABI2 in Model2 compared to Model1. This is because Ca^2+^_c_ can never be in fixed ON state due to the Ca^2+^_c_ – Ca^2+^ ATPase negative feedback; at best, it can oscillate. As a result, in Model2, the inhibition of ABI2 can also be oscillatory, i.e., ABI2 is OFF whenever Ca^2+^_c_ is ON but it is free to be ON when Ca^2+^_c_ oscillation is in the OFF state and its other regulator, ROS, has not yet stabilized.

Let us now consider the case of Model2 where the nodes CPK3/21, MPK9/12, Vacuolar Acidification, and Microtubule Depolymerization are regulated by the cumulative effect of the past states of their respective regulator nodes. The succession diagram of this model version with short-term memory in the absence of ABA is independent of memory. The absence of ABA leads to four stable motifs which are the same as *openM1*, *openM2*, *openM3*, and *openM4*, detailed in Figures 2 and S2. Since any pair of these four motifs overlap in nodes, they cannot be on the same trajectory. Hence, the succession diagram has four trajectories, where each corresponds to each of these motifs leading to an open stomata attractor. The attractor reached by any of these motifs is the same. This attractor is also the same as the one listed in the third part of Table S4. Figure S3 shows the complete succession diagram for this case. The succession diagram of Model2 with short-term memory in the presence of ABA is identical to the succession diagram of Model1 with short-term memory in the presence of ABA – see Figure 8B.


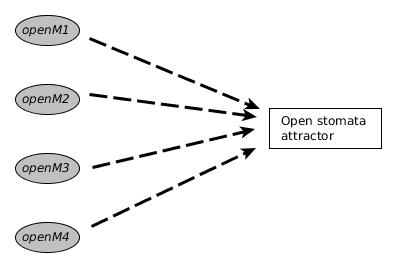


**Figure S3. Complete succession diagram for Model2 in the absence of ABA for the model version with short term memory.** Assuming the absence of any signal, this model always leads to an open stomata attractor.

**Text S3. Complete Boolean update functions implementing short term memory rules of the network simulation shown in Figure 7.**

Ca_1 = Ca_2 = Ca_3 = TCTP_1 = TCTP_2 = TCTP_3 = TCTP_4 = TCTP_5 = VATPase_1 = VATPase_2 = Ca = ABA = Closure = ROP11 = H2O_Efflux = Microtubule_Depolymerization = Ca_ATPase = RCARs = RBOH = pHc = NO = CaIM = K_efflux = CIS = Vacuolar_Acidification = OST1 = SPHK12 = PLDa = PA = NIA12 = NOGC1 = Actin = InsP3 = CPKa = MPK = False

Malate = H_ATPase = ABI1 = ABI2 = HAB1 = PP2CA = CPKb = True

1: TCTP_5* = TCTP_4

2: TCTP_4* = TCTP_3

3: TCTP_3* = TCTP_2

4: TCTP_2* = TCTP_1

5: TCTP_1* = TCTP

1: VATPase_2* = VATPase_1

2: VATPase_1* = VATPase

1: Ca_3* = Ca_2

2: Ca_2* = Ca_1

3: Ca_1* = Ca

6: RCARs* = ABA

6: PEPC* = not ABA

6: PI3P5K* = ABA

6: ABI1* = not PA and (not RCARs or ROP11) and not RBOH and pHc

6: ABI2* = (not RCARs or ROP11) and not RBOH and not PA

6: HAB1* = not RCARs and not RBOH

6: PP2CA* = not RCARs and not RBOH

6: OST1* = (not ABI1 and not HAB1) or (not PP2CA and not ABI2) or (not ABI1 and not ABI2) or (not HAB1 and not PP2CA) or (not HAB1 and not ABI2) or (not ABI1 and not PP2CA)

6: RBOH* = pHc and not ABI1 and OST1 and SPHK12 and PA

6: GHR1* = not ABI2 and RBOH

6: NO* = NIA12

6: NIA12* = RBOH

6: NOGC1* = NO

6: nitrocGMP* = NOGC1 and RBOH and NO

6: CIS* = InsP3 or nitrocGMP

6: CaIM* = Actin or GHR1

6: Ca* = (CIS or CaIM) and not Ca_ATPase

6: Ca_ATPase* = Ca

6: CPKa* = Ca or Ca_1 or Ca_2 or Ca_3

6: MPK* = Ca or Ca_1 or Ca_2 or Ca_3

6: PLC* = Ca

6: DAG* = PLC

6: InsP3* = PLC

6: PLDa* = SPHK12 and Ca

6: PLDdel* = NO or RBOH

6: PA* = PLDdel or PLDa or DAG

6: SPHK12* = PA or ABA

6: VATPase* = Ca

6: TCTP* = Ca

6: Microtubule_Depolymerization* = TCTP or TCTP_1 or TCTP_2 or TCTP_3 or TCTP_4 or TCTP_5

6: pHc* = ((OST1 and not ABI2 and not ABI1) or Ca) and Vacuolar_Acidification

6: H_ATPase* = not pHc and not Ca and not RBOH

6: AtRAC1* = not ABA or ABI1

6: Actin* = not AtRAC1

6: SLAC1* = (CPKb or CPKa) and MPK and OST1 and GHR1 and not ABI1 and not PP2CA and not ABI2 and pHc

6: QUAC1* = OST1 and Ca

6: SLAH3* = CPKb and CPKa and not ABI1

6: AnionEM* = SLAC1 or QUAC1 and SLAH3

6: Malate* = PEPC and not ABA and not AnionEM

6: KEV* = Vacuolar_Acidification or Ca

6: Depolarization* = (AnionEM or Ca or KEV) and (not H_ATPase or not K_efflux)

6: KOUT* = (not NO or not RBOH or pHc) and Depolarization

6: K_efflux* = KEV and KOUT

6: H2O_Efflux* = AnionEM and OST1 and K_efflux and not Malate

6: Closure* = Microtubule_Depolymerization and H2O_Efflux

6: Vacuolar_Acidification* = PI3P5K or VATPase or VATPase_1 or VATPase_2

**Text S4. Analysis of different short-term memory durations.**

1. The probability of a node state being ON as a function of the time window of memory.

As described in section 3.2 of the main text, we consider four nodes in the network whose sustained activity was previously modeled by self-loops and replace them with short term memory of their regulator. This short-term memory aims to ensure that the target node can stabilize in the ON state if the regulator has sustained, high-frequency oscillations; when the oscillations cease, the target node stabilizes in the OFF state. This is important so that the network model recapitulates the reopening of the stomata after the signal is removed. These four nodes are CPK3/21, MPK9/12, Microtubule Depolymerization, and Vacuolar Acidification. As shown in Figure 6, two of these nodes, CPK3/21 and MPK9/12, are directly regulated by Ca^2+^_c_ while the other two, Microtubule Depolymerization and Vacuolar Acidification, are two edges away from Ca^2+^_c_. Here we determine the probability of observing an ON state of a target node at distance one or two from Ca^2+^_c_ as a function of the duration of the short-term memory.

We consider the regulation of Ca^2+^_c_ after a sustained ON state of CIS or CaIM has been established (e.g. by ABA). In this situation Ca^2+^_c_ and Ca^2+^ ATPase form a two-node negative feedback loop. We also consider a target node regulated (directly or through a mediator) by Ca^2+^_c_. We simulate this system using the rank order asynchronous update in BooleanNet (see Section 3.2 for more details). For simplicity we use the notation A for Ca^2+^_c_ and B for Ca^2+^ ATPase. The set of rules for one timestep memory and regulator-target distance of one (d=1) is given below. A_1 represents A_t-1_.

rank 1 (updated first): A_1* = A
rank 2: A* = not B
rank 2: B* = A
rank 2: C* = A or A_1

We determine the probability of the ON state of the target node as the duration of the short-term memory is increased (see Figure S4).


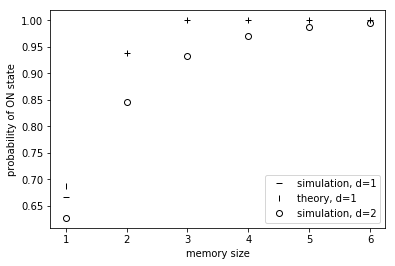


**Figure S4. The probability of an oscillation-driven target node being ON as a function of its short-term memory duration, determined analytically and by simulation.** Horizontal lines represent the ON probability of a node that is one edge away from the oscillating node (d = 1) while vertical lines represent the probabilities calculated analytically. Circles represent the ON probability of a node that is two edges away from the oscillating node (d = 2).

We can analyze the state transition graph of a two-node negative feedback loop to understand the dependence of the probability of the ON state on the memory duration. The regulatory network of A and B is given in Figure S5A and the state transition diagram that results from considering the dynamical system of these two nodes is given in Figure S5B.


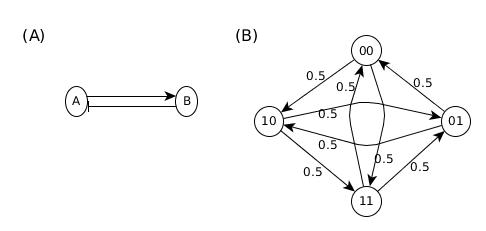


**Figure S5. Analysis of a toy network representing the oscillation of Ca^2+^_c_ in the negative feedback loop formed by Ca^2+^_c_ and Ca^2+^ ATPase.** (A) Network diagram, where node A represents Ca^2+^_c_ and B represents Ca^2+^ ATPase. Hence, the update rules are: A* = not B; B* = A. (B) State transition graph for the two-node toy network in the random order asynchronous update scheme (the update scheme used in our analysis of the ABA network). Each state is represented in the A,B order of nodes*.*

Consider the case when the memory duration is 1, so the update rule for the target node C is: C*= A_t_ or A_t-1_ where t is the current time. Since this is random order asynchronous update, in 50% of the update orders, C will be updated before A and in the other 50% of the update orders, A will be updated before C. So, we can calculate the overall probability of the ON state of C as follows:

P(C=ON) = 0.5*P(C=1 if C is updated before A) + 0.5*P(C=1 if A is updated before C)
 *If C is updated before A, A_t_ = A_t-1_, hence the state of C only depends on A _t-1_*

= 0.5*P(A_t-1_=1) + 0.5*(1-P(C=0 if A is updated before C))

*For C = A_t_ or A_t-1_ to be 0, both A_t_ and A_t-1_ should be 0*

= 0.5*0.5 + 0.5*(1-P(A_t-1_=A_t_=0))

*From Bayes’ theorem, P(A_t_=0 & A_t-1_=0) = P(A_t-1_=0)*P(A_t_=0|A_t-1_=0)*

= 0.25 + 0.5*(1-(P(A_t-1_=0)*P(A_t_=0|A_t-1_=0)))

*From the symmetry of the state transition diagram in Figure S5B, the A,B system can be in any of the 00, 01, 10, 11 states with equal likelihood at t-1. Of these four states A_t-1_ = 0 in 2 states hence, P(A_t-1_=0) = 0.5. When we are given that A_t-1_ = 0, the system is either in 00 or 01 with equal likelihood. From the 00 state the system can go to 11 or 10; from the 01 state the system can go to 10 or 00. This gives us four possible, equally likely trajectories, i.e.,* ***0****0→****1****1;* ***0****0→****1****0;* ***0****1→****1****0;* ***0****1→****0****0. (The state of node A is marked in bold). Of these four trajectories, only the 01→00 trajectory corresponds to A_t_=0. Hence, P(A_t_=0|A_t-1_=0)=0.25.*

= 0.25 + 0.5*(1-(0.5*0.25))

= 0.6875

Similarly, we can work out the case when the memory duration is 2 and hence the update rule for the target node C is: C* = A_t_ or A_t-1_ or A_t-2_. We again consider two equally likely scenarios, one where C is updated before A and the other where A is updated before C. In this case as well, we can calculate the overall probability of the ON state of C as follows:

P(C=ON) = 0.5* P(C=1 if C is updated before A) + 0.5*P(C=1 if A is updated before C)

*The probability that C is ON can be calculated by subtracting the probability that C is OFF from 1.*

= 0.5* (1-P(C=0 if C is updated before A)) + 0.5*(1-P(C=0 if A is updated before C))

*If C is updated before A, the state A_t_ = A_t-1_ hence for C=0, we only need that A_t-2_=A_t-1_=0. But if A is updated before C, we need that each of A_t-2_, A_t-1_, and A_t_ are 0 for C=0.*

= 0.5*(1-P(A_t-2_=A_t-1_=0)) + 0.5*(1-P(A_t-2_=A_t-1_=A_t_=0))

*From the previous calculation, the probability that the state of A is 0 for two consecutive timesteps can be calculated using Bayes’ theorem and it results in 0.5*0.25 = 0.125. The state of A cannot stay 0 for three consecutive timesteps. The state of A is 0 for two consecutive timesteps only for the trajectory* ***0****1→****0****0. When the system is in 00 state, it can only go to 11 or 10. Hence, A_t-2_=A_t-1_=A_t_=0 is impossible.*

= 0.5*(1-0.125) + 0.5*(1-0)

= 0.9375

Extending the same logic, we can calculate the probability of C turning ON when considering a memory duration of 3. In this case, the update rule is: C* = A_t_ or A_t-1_ or A_t-2_ or A_t-3_. There are again two equally likely scenarios, one where C is updated before A and other where A is updated before C. Hence, the probability calculations are as follows:

P(C=ON) = 0.5* P(C=1 if C is updated before A) + 0.5*P(C=1 if A is updated before C)

*The probability that C is ON can be calculated by subtracting the probability that C is OFF from 1.*

= 0.5* (1-P(C=0 if C is updated before A)) + 0.5*(1-P(C=0 if A is updated before C))

*If C is updated before A, the state A_t_ = A_t-1_ hence for C=0, we only need that A_t-3_=A_t-2_=A_t-1_=0. But if A is updated before C, we need that each of A_t-3_, A_t-2_, A_t-1_, and A_t_ are 0 for C=0.*

= 0.5*(1-P(A_t-3_=A_t-2_=A_t-1_=0)) + 0.5*(1-P(A_t-3_=A_t-2_=A_t-1_=A_t_=0))

*In the previous calculation, we saw that it is impossible for A to be 0 for three consecutive timesteps. Hence, it is also impossible for A to be 0 for four consecutive timesteps.*

= 0.5*(1-0) + 0.5*(1-0)

= 1.0

Hence, the probability of the ON state of the target node is 1 when the memory duration is 3 or more. As we can see in Figure S4, the calculated probabilities and the probabilities obtained from simulation are very close for memory of one time step and coincide with each other for larger memory duration. Following the same method described above, one can calculate the probability of the ON state for Vacuolar Acidification and Microtubule Depolymerization, i.e., nodes at distance 2 using the state transition diagram for three nodes given in Figure S3 of Maheshwari et al. (2019). As the corresponding state transition diagram has multiple cycles, the calculation is complex, and we skip reproducing it here. Figure S4 indicates that a target node regulated by the Ca^2+^_c_ – Ca^2+^ ATPase negative feedback loop is in a sustained ON state with a probability of 100% if it considers the last three timesteps, and a target node two steps away from the feedback loop is ON with probability >95% if it considers the last five timesteps.

1. Choosing the optimal memory duration of each of the nodes.

The choice of the memory duration for each node in consideration determines the final percentage of closure the network can reach in the sustained presence of a signal and the rate of reopening after the signal is removed. Here we determine the constraints on the memory duration of each of the four nodes and summarize the results in Table S8. Of the four nodes in consideration, CPK3/21 and MPK9/12 affect closure by affecting anion channels; anion flow is a necessary contributor to water efflux, which is necessary for closure. Microtubule Depolymerization is another necessary contributor to closure. From Figure S4 we can see that for nodes that are one edge away from Ca^2+^_c_, i.e., CPK3/21 and MPK9/12, memory duration of 3 is enough to ensure persistence of these nodes and a close to 100% final percentage of closure. Microtubule Depolymerization has a stronger effect on the final percentage of closure, so a memory duration of 5 or more timesteps is necessary to ensure >97% persistence of Microtubule Depolymerization and ~100% final percentage of closure. There are no constraints on CPK3/21, MPK9/12 and Microtubule Depolymerization regarding reopening.

Vacuolar Acidification is necessary for pH_c_ and ROS, so its ON state is an important prerequisite for the establishment and subsequent stabilization of the *closureM* motif. In the presence of ABA, Vacuolar Acidification is sustained in the ON state since ABA is a sufficient and necessary regulator of V-PPase, which is a sufficient regulator of Vacuolar Acidification. Hence the memory duration for Vacuolar Acidification does not affect the closure induced by ABA. During closure induced by external Ca^2+^, oscillations of Ca^2+^_c_ regulate Vacuolar Acidification, thus a sufficiently large memory duration is crucial in ensuring that Vacuolar Acidification is sustained in the ON state, enabling the establishment of the *closureM* motif. During reopening following the removal of a closure-inducing signal, Vacuolar Acidification must be OFF (in order to destabilize the *closureM* motif). Thus, a smaller memory duration yields faster reopening.

**Table S8**. **Summary of the constraints on the memory duration for each of the four nodes in order for the model to reproduce stomatal closure in response to ABA or external Ca^2+^ as well as stomatal reopening following the removal of the signal.** We consider four scenarios: stomatal closure induced by the sustained presence of ABA (second column), reopening of the stomata following removal of ABA (third column), stomatal closure induced by external Ca^2+^ (fourth column), and the reopening of the stomata following removal of external Ca^2+^ (fifth column). Memory durations that satisfy the constraints of all four scenarios (for example, a memory of three or more time steps for CPK3/21) simultaneously ensure efficient closure and reopening. This sensitivity analysis of the memory durations of nodes leads to the conclusion that the best case has memory sizes of 3, 3, 5, for CPK3/21, MPK9/12, and Microtubule Depolymerization, respectively. For Vacuolar Acidification, memory size of 2 or 3 could be equally good.

| Node | ABA induced closure | Reopening after ABA removal | External Ca^2+^ induced closure | Reopening after external Ca^2+^ removal |
| --- | --- | --- | --- | --- |
| CPK3/21 | ≥ 3 | no constraint | ≥ 3 | no constraint |
| MPK9/12 | ≥ 3 | no constraint | ≥ 3 | no constraint |
| Microtubule Depolymerization | ≥ 5 | no constraint | ≥ 5 | no constraint |
| Vacuolar Acidification | no constraint | ≤ 3 | ≥ 2 | ≤ 3 |

In summary, the choice of memory duration for Vacuolar Acidification involves a tradeoff. A larger memory duration would mean larger final percentage of closure in the presence of a closure signal other than ABA, while smaller memory duration would mean faster reopening when the signal is removed; both effects are desirable. Hence, we tried two values of memory duration for Vacuolar Acidification: 2 and 3. After simulating external calcium-induced closure (using CaIM=1) for both these values, we find that memory duration of 2 gives ~70% closure and memory duration of 3 gives ~85% closure. A simulation of ABA-induced closure gives ~100% stomatal closure for memory duration of 2 as well as memory duration of 3. However, the reopening is much slower with either signal (ABA or external Ca^2+^) when the memory duration is 3, hence, we pick the case with Vacuolar Acidification memory duration of 2 as the most appropriate option. Figures 7 and S6 show the effect of signal addition and removal on this network where the signal is ABA or external Ca^2+^ respectively.


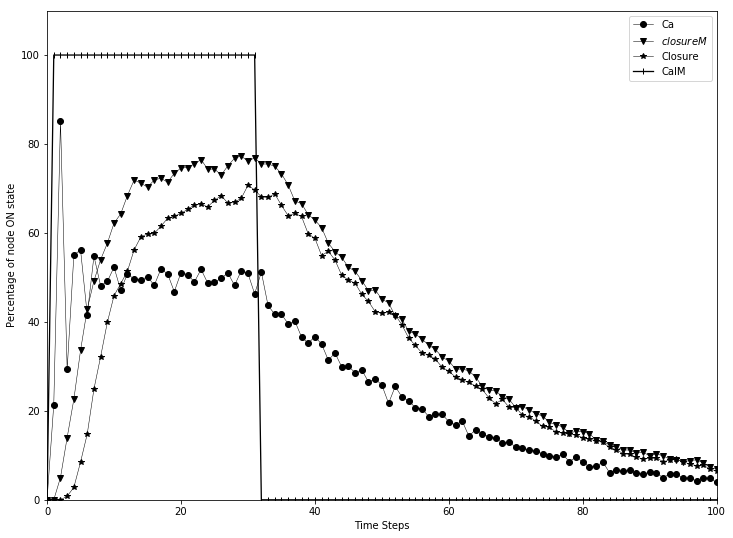


**Figure S6. Simulation of the model with short term memory when external Calcium is present for the first 30 timesteps and absent for the following 70 timesteps.** The presence or absence of external Calcium is simulated by the ON or OFF state of CaIM respectively. The memory duration is 3 for CPK3/21 and MPK9/12, 5 for Microtubule Depolymerization and 2 for Vacuolar Acidification. The ON state of CaIM leads to ~70% stomatal closure; after CaIM is set to OFF state, the percentage of closure reduces to ~30% within 30 timesteps.

In the case we determine as optimal, presented in Figures 7 and S6, the peak percentage of closure is different for different signals. The fixed ON state of ABA leads to ~100% closure while the fixed ON state of CaIM leads to ~70% closure in 30 timesteps, which is the same as the maximum it can reach with the sustained presence of CaIM. As shown in Figure S7, if the memory of Vacuolar Acidification is two time steps, it can achieve ~85% closure, and lead to the activation of the *closureM* motif in approximately 70% of simulations.

**
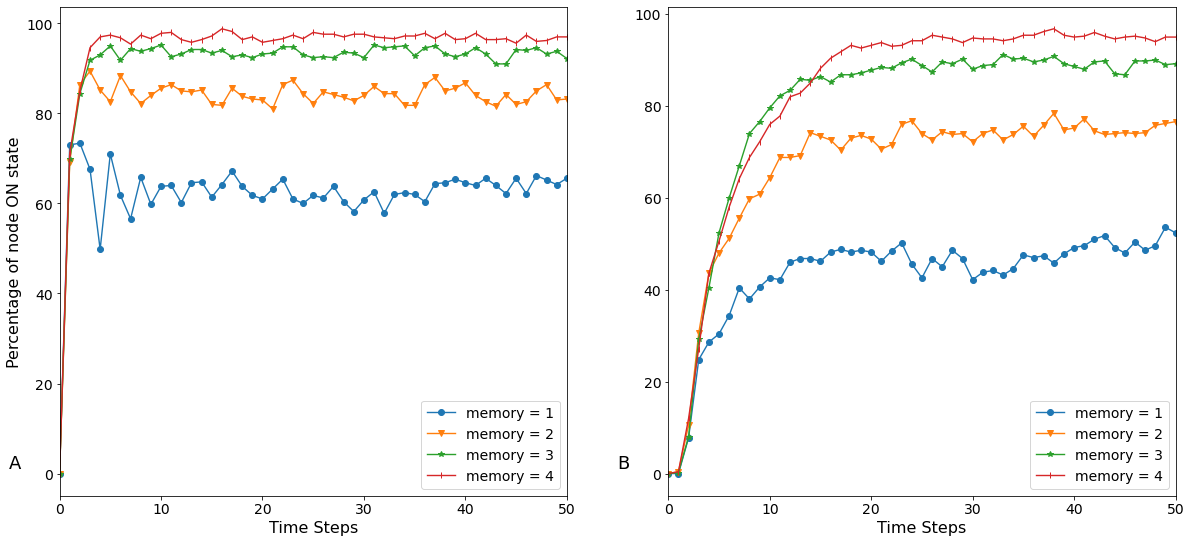
**

**Figure S7. Effect of the memory duration on the percentage of the ON state of the Vacuolar Acidification node and the stabilization of the *closureM* motif.** **A**. The time-course of the percentage of ON state of the Vacuolar Acidification node for different memory durations from 1 to 4 time-steps. **B**. The time-course of the percent of ON state (= stabilization) of the *closureM* stable motif for different memory durations from1 to 4. When there is no memory, Vacuolar Acidification oscillates as Ca^2+^_c_ does and hence its average activity stays at 50%. With memory duration=1, Vacuolar Acidification percentage is greater than 50% and it increases progressively with increased memory duration. The ON state of Vacuolar Acidification is necessary for the *closureM* motif and hence at any memory duration, the percent stabilization of *closureM* is always less than the percent of the ON state of Vacuolar Acidification.

**Text S5. Bifurcation analysis of the guard cell model.**

Bifurcation diagrams of continuous systems plot the steady state values of a response variable for the whole range of a continuous parameter. This is not exactly replicable in Boolean models, which by default do not have numerical parameters, and whose variables have just two values. Nevertheless, the previous and current analysis of the guard cell model does include the identification of the attractor(s) in the presence or absence of ABA and for the knockout or constitutive activation of each node in turn. The results of this analysis indicate that the unperturbed system in the presence of ABA has a single attractor, which corresponds to stomatal closure. Knockout of a node in the presence of ABA may preserve the attractor, it may lead to an attractor in which Closure oscillates, or it may lead to an attractor that corresponds to open stomata. The nodes in each category are indicated in Table 3 in Albert et al. (2017).

In the absence of ABA, the unperturbed system is multi-stable, admitting 16 very similar attractors that correspond to open stomata and an attractor that corresponds to stomatal closure. Constitutive activation of a node may preserve the multi-stability of the system or it may lead to the closure attractor to become the sole attainable attractor; see Table 2 in Maheshwari et al. (2019). Under a sequence of ABA=off, then ABA=on, then ABA=off again, the original model behaves as an irreversible switch: the closure attractor is not reversible by turning ABA off (see red trajectory in Figure S8). The model with short-term memory makes the switch reversible (see blue trajectory in Figure S8). The (conditionally) stable motifs provide the explanation of these findings. In the model that assumes the persistent activity of CPK3/21, MPK9/12, Microtubule depolymerization and Vacuolar acidification, each of these four nodes forms a stable motif regardless of the status of ABA, thus they stay ON following the removal of ABA, which in turn maintains the activation of the *closureM* motif and thus maintains closure (see Section 3.2). In the model with short-term memory these four nodes do not form stable motifs. The loss of ABA yields the successive decay of Ca^2+^_c_ oscillations and of the *closureM* motif, followed by the establishment of the *openM1* motif and convergence to the open stomata attractor (see Section 3.3.2).


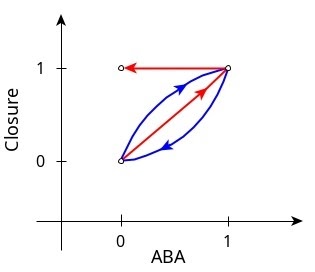
**Figure S8. The Boolean bifurcation diagram of the model for the two assumptions regarding the memory for CPK3/21, MPK9/12, Microtubule depolymerization and Vacuolar acidification.** The red lines represent the network model in Albert et al. (2017); Maheshwari et al. (2019), which assumes persistent activity, i.e., infinite memory for these nodes. In this model, the presence of ABA leads to the attractor corresponding to stomatal closure. After the removal of ABA, a few nodes change state but the stable motifs of the system remain locked in and the corresponding attractor still has the node Closure in the ON (1) state. The blue lines represent the updated network model with short-term memory. The presence of ABA leads to stomatal closure (in a slightly different trajectory) and the removal of ABA leads to reopening of the stomata.

References:

Acharya, Biswa R., Byeong Wook Jeon, Wei Zhang, and Sarah M. Assmann. 2013. “Open Stomata 1 (OST 1) Is Limiting in Abscisic Acid Responses of Arabidopsis Guard Cells.” *New Phytologist* 200 (4): 1049–1063.

Albert, Réka, Biswa R Acharya, Byeong Wook Jeon, Jorge GT Zañudo, Mengmeng Zhu, Karim Osman, and Sarah M Assmann. 2017. “A New Discrete Dynamic Model of ABA-Induced Stomatal Closure Predicts Key Feedback Loops.” *PLoS Biology* 15 (9): e2003451.

Antoni, Regina, Miguel Gonzalez-Guzman, Lesia Rodriguez, Americo Rodrigues, Gaston A. Pizzio, and Pedro L. Rodriguez. 2012. “Selective Inhibition of Clade A Phosphatases Type 2C by PYR/PYL/RCAR Abscisic Acid Receptors.” *Plant Physiology* 158 (2): 970–980.

Bak, Gwangbae, Eun-Jung Lee, Yuree Lee, Mariko Kato, Shoji Segami, Heven Sze, Masayoshi Maeshima, Jae-Ung Hwang, and Youngsook Lee. 2013. “Rapid Structural Changes and Acidification of Guard Cell Vacuoles during Stomatal Closure Require Phosphatidylinositol 3, 5-Bisphosphate.” *The Plant Cell* 25 (6): 2202–2216.

Boss, Wendy F., and Yang Ju Im. 2012. “Phosphoinositide Signaling.” *Annual Review of Plant Biology* 63: 409–429.

Bright, Jo, Radhika Desikan, John T. Hancock, Iain S. Weir, and Steven J. Neill. 2006. “ABA-Induced NO Generation and Stomatal Closure in Arabidopsis Are Dependent on H_2_O_2_ Synthesis.” *The Plant Journal* 45 (1): 113–122.

Coursol, Sylvie, Liu-Min Fan, Hervé Le Stunff, Sarah Spiegel, Simon Gilroy, and Sarah M Assmann. 2003. “Sphingolipid Signalling in Arabidopsis Guard Cells Involves Heterotrimeric G Proteins.” *Nature* 423 (6940): 651–654.

Desikan, Radhika, Rachael Griffiths, John Hancock, and Steven Neill. 2002. “A New Role for an Old Enzyme: Nitrate Reductase-Mediated Nitric Oxide Generation Is Required for Abscisic Acid-Induced Stomatal Closure in Arabidopsis Thaliana.” *Proceedings of the National Academy of Sciences* 99 (25): 16314–16318.

Distefano, Ayelen M., CARLOS GARCÍA-MATA, Lorenzo Lamattina, and Ana M. Laxalt. 2008. “Nitric Oxide-Induced Phosphatidic Acid Accumulation: A Role for Phospholipases C and D in Stomatal Closure.” *Plant, Cell & Environment* 31 (2): 187–194.

Distéfano, Ayelen M., Denise Scuffi, Carlos García-Mata, Lorenzo Lamattina, and Ana M. Laxalt. 2012. “Phospholipase Dδ Is Involved in Nitric Oxide-Induced Stomatal Closure.” *Planta* 236 (6): 1899–1907.

Dittrich, P., and K. Raschke. 1977. “Malate Metabolism in Isolated Epidermis of Commelina Communis L. in Relation to Stomatal Functioning.” *Planta* 134 (1): 77–81.

Du, Zhirong, Karthik Aghoram, and William H. Outlaw Jr. 1997. “In VivoPhosphorylation of Phosphoenolpyruvate Carboxylase in Guard Cells OfVicia FabaL. Is Enhanced by Fusicoccin and Suppressed by Abscisic Acid.” *Archives of Biochemistry and Biophysics* 337 (2): 345–350.

Gardner, Melissa K., Marija Zanic, and Jonathon Howard. 2013. “Microtubule Catastrophe and Rescue.” *Current Opinion in Cell Biology* 25 (1): 14–22.

Geiger, Dietmar, Tobias Maierhofer, Khaled AS AL-Rasheid, Sönke Scherzer, Patrick Mumm, Anja Liese, Peter Ache, Christian Wellmann, Irene Marten, and Erwin Grill. 2011. “Stomatal Closure by Fast Abscisic Acid Signaling Is Mediated by the Guard Cell Anion Channel SLAH3 and the Receptor RCAR1.” *Science Signaling* 4 (173): ra32–ra32.

Geiger, Dietmar, Sönke Scherzer, Patrick Mumm, Annette Stange, Irene Marten, Hubert Bauer, Peter Ache, Susanne Matschi, Anja Liese, and Khaled AS Al-Rasheid. 2009. “Activity of Guard Cell Anion Channel SLAC1 Is Controlled by Drought-Stress Signaling Kinase-Phosphatase Pair.” *Proceedings of the National Academy of Sciences* 106 (50): 21425–21430.

Gonugunta, Vijay K, Nupur Srivastava, Mallikarjuna R Puli, and Agepati S Raghavendra. 2008. “Nitric Oxide Production Occurs after Cytosolic Alkalinization during Stomatal Closure Induced by Abscisic Acid.” *Plant, Cell & Environment* 31 (11): 1717–1724.

Grondin, Alexandre, Olivier Rodrigues, Lionel Verdoucq, Sylvain Merlot, Nathalie Leonhardt, and Christophe Maurel. 2015. “Aquaporins Contribute to ABA-Triggered Stomatal Closure through OST1-Mediated Phosphorylation.” *The Plant Cell* 27 (7): 1945–1954.

Guo, L., and X. Wang. 2012. “Crosstalk between Phospholipase D and Sphingosine Kinase in Plant Stress Signaling.” *Frontiers in Plant Science* 3: 51–51.

Guo, Liang, Shivakumar P. Devaiah, Rama Narasimhan, Xiangqing Pan, Yanyan Zhang, Wenhua Zhang, and Xuemin Wang. 2012. “Cytosolic Glyceraldehyde-3-Phosphate Dehydrogenases Interact with Phospholipase Dδ to Transduce Hydrogen Peroxide Signals in the Arabidopsis Response to Stress.” *The Plant Cell* 24 (5): 2200–2212.

Guo, Liang, Girish Mishra, Kyle Taylor, and Xuemin Wang. 2011. “Phosphatidic Acid Binds and Stimulates Arabidopsis Sphingosine Kinases.” *Journal of Biological Chemistry* 286 (15): 13336–13345.

Guse, Andreas H. 1999. “Cyclic ADP-Ribose: A Novel Ca^2+^-Mobilising Second Messenger.” *Cellular Signalling* 11 (5): 309–316.

Hedrich, Rainer. 2012. “Ion Channels in Plants.” *Physiological Reviews*.

Hosy, Eric, Alain Vavasseur, Karine Mouline, Ingo Dreyer, Frédéric Gaymard, Fabien Porée, Jossia Boucherez, et al. 2003. “The Arabidopsis Outward K^+^ Channel GORK Is Involved in Regulation of Stomatal Movements and Plant Transpiration.” *Proceedings of the National Academy of Sciences* 100 (9): 5549–5554.

Hua, Deping, Cun Wang, Junna He, Hui Liao, Ying Duan, Ziqiang Zhu, Yan Guo, Zhizhong Chen, and Zhizhong Gong. 2012. “A Plasma Membrane Receptor Kinase, GHR1, Mediates Abscisic Acid-and Hydrogen Peroxide-Regulated Stomatal Movement in Arabidopsis.” *The Plant Cell* 24 (6): 2546–2561.

Imes, Dennis, Patrick Mumm, Jennifer Böhm, Khaled AS Al-Rasheid, Irene Marten, Dietmar Geiger, and Rainer Hedrich. 2013. “Open Stomata 1 (OST 1) Kinase Controls R–Type Anion Channel QUAC 1 in A Rabidopsis Guard Cells.” *The Plant Journal* 74 (3): 372–382.

Islam, Mohammad Mahbub, Mohammad Anowar Hossain, Rayhanur Jannat, Shintaro Munemasa, Yoshimasa Nakamura, Izumi C. Mori, and Yoshiyuki Murata. 2010. “Cytosolic Alkalization and Cytosolic Calcium Oscillation in Arabidopsis Guard Cells Response to ABA and MeJA.” *Plant and Cell Physiology* 51 (10): 1721–1730.

Jiang, Yan, Kai Wu, Feng Lin, Yana Qu, Xiaoxiang Liu, and Qun Zhang. 2014. “Phosphatidic Acid Integrates Calcium Signaling and Microtubule Dynamics into Regulating ABA-Induced Stomatal Closure in Arabidopsis.” *Planta* 239 (3): 565–575.

Joudoi, Takahiro, Yudai Shichiri, Nobuto Kamizono, Takaaki Akaike, Tomohiro Sawa, Jun Yoshitake, Naotaka Yamada, and Sumio Iwai. 2013. “Nitrated Cyclic GMP Modulates Guard Cell Signaling in Arabidopsis.” *The Plant Cell* 25 (2): 558–571.

Kim, Yong-Min, Yun-Jeong Han, Ok-Jin Hwang, Si-Seok Lee, Ah-Young Shin, Soo Young Kim, and Jeong-Il Kim. 2012. “Overexpression of Arabidopsis Translationally Controlled Tumor Protein Gene AtTCTP Enhances Drought Tolerance with Rapid ABA-Induced Stomatal Closure.” *Molecules and Cells* 33 (6): 617–626.

Kinoshita, Toshinori, Mitsuo Nishimura, and K. I. Shimazaki. 1995. “Cytosolic Concentration of Ca^2+^ Regulates the Plasma Membrane H^+^-ATPase in Guard Cells of Fava Bean.” *The Plant Cell* 7 (8): 1333–1342.

Köhler, Barbara, Adrian Hills, and Michael R. Blatt. 2003. “Control of Guard Cell Ion Channels by Hydrogen Peroxide and Abscisic Acid Indicates Their Action through Alternate Signaling Pathways.” *Plant Physiology* 131 (2): 385–388.

Kwak, June M., Ji-Hye Moon, Yoshiyuki Murata, Kazuyuki Kuchitsu, Nathalie Leonhardt, Alison DeLong, and Julian I. Schroeder. 2002. “Disruption of a Guard Cell–Expressed Protein Phosphatase 2A Regulatory Subunit, RCN1, Confers Abscisic Acid Insensitivity in Arabidopsis.” *The Plant Cell* 14 (11): 2849–2861.

Lemichez, Emmanuel, Yan Wu, Juan-Pablo Sanchez, Amel Mettouchi, Jaideep Mathur, and Nam-Hai Chua. 2001. “Inactivation of AtRac1 by Abscisic Acid Is Essential for Stomatal Closure.” *Genes & Development* 15 (14): 1808–1816.

Lemtiri-Chlieh, Fouad, Enid AC MacRobbie, Alex AR Webb, Nick F. Manison, Colin Brownlee, Jeremy N. Skepper, Jian Chen, Glenn D. Prestwich, and Charles A. Brearley. 2003. “Inositol Hexakisphosphate Mobilizes an Endomembrane Store of Calcium in Guard Cells.” *Proceedings of the National Academy of Sciences* 100 (17): 10091–10095.

Leube, M. P., E. Grill, and N. Amrhein. 1998. “ABI1 of Arabidopsis Is a Protein Serine/Threonine Phosphatase Highly Regulated by the Proton and Magnesium Ion Concentration 1.” *FEBS Letters* 424 (1–2): 100–104.

Li, Song, Sarah M Assmann, and Réka Albert. 2006. “Predicting Essential Components of Signal Transduction Networks: A Dynamic Model of Guard Cell Abscisic Acid Signaling.” *PLoS Biology* 4 (10).

Li, Zixing, Zheng Li, Xiang Gao, Viswanathan Chinnusamy, Ray Bressan, Zhi-Xin Wang, Jian-Kang Zhu, Jia-Wei Wu, and Dong Liu. 2012. “ROP11 GTPase Negatively Regulates ABA Signaling by Protecting ABI1 Phosphatase Activity from Inhibition by the ABA Receptor RCAR1/PYL9 in Arabidopsis.” *Journal of Integrative Plant Biology* 54 (3): 180–188.

Luo, Hong, Pierre Morsomme, and Marc Boutry. 1999. “The Two Major Types of Plant Plasma Membrane H^+^-ATPases Show Different Enzymatic Properties and Confer Differential PH Sensitivity of Yeast Growth.” *Plant Physiology* 119 (2): 627–634.

Ma, Yue, Izabela Szostkiewicz, Arthur Korte, Danièle Moes, Yi Yang, Alexander Christmann, and Erwin Grill. 2009. “Regulators of PP2C Phosphatase Activity Function as Abscisic Acid Sensors.” *Science* 324 (5930): 1064–1068.

Maheshwari, Parul, Hao Du, Jen Sheen, Sarah M Assmann, and Reka Albert. 2019. “Model-Driven Discovery of Calcium-Related Protein-Phosphatase Inhibition in Plant Guard Cell Signaling.” *PLoS Computational Biology* 15 (10).

Martinoia, Enrico, Masayoshi Maeshima, and H. Ekkehard Neuhaus. 2007. “Vacuolar Transporters and Their Essential Role in Plant Metabolism.” *Journal of Experimental Botany* 58 (1): 83–102.

Meinhard, Michael, and Erwin Grill. 2001. “Hydrogen Peroxide Is a Regulator of ABI1, a Protein Phosphatase 2C from Arabidopsis.” *Febs Letters* 508 (3): 443–446.

Meinhard, Michael, Pedro L. Rodriguez, and Erwin Grill. 2002. “The Sensitivity of ABI2 to Hydrogen Peroxide Links the Abscisic Acid-Response Regulator to Redox Signalling.” *Planta* 214 (5): 775–782.

Merlot, Sylvain, Françoise Gosti, Danièle Guerrier, Alain Vavasseur, and Jérôme Giraudat. 2001. “The ABI1 and ABI2 Protein Phosphatases 2C Act in a Negative Feedback Regulatory Loop of the Abscisic Acid Signalling Pathway.” *The Plant Journal* 25 (3): 295–303.

Miedema, H., and Sarah M. Assmann. 1996. “A Membrane-Delimited Effect of Internal pH on the K^+^ Outward Rectifier of Vicia Faba Guard Cells.” *The Journal of Membrane Biology* 154 (3): 227–237.

Mulaudzi, Takalani, Ndiko Ludidi, Oziniel Ruzvidzo, Monique Morse, Nicolette Hendricks, Emmanuel Iwuoha, and Chris Gehring. 2011. “Identification of a Novel Arabidopsis Thaliana Nitric Oxide-Binding Molecule with Guanylate Cyclase Activity in Vitro.” *FEBS Letters* 585 (17): 2693–2697.

Munnik, T., R. F. Irvine, and A. Musgrave. 1998. “Phospholipid Signalling in Plants.” *Biochimica et Biophysica Acta (BBA)-Lipids and Lipid Metabolism* 1389 (3): 222–272.

Murata, Yoshiyuki, Zhen-Ming Pei, Izumi C Mori, and Julian Schroeder. 2001. “Abscisic Acid Activation of Plasma Membrane Ca^2+^ Channels in Guard Cells Requires Cytosolic NAD(P)H and Is Differentially Disrupted Upstream and Downstream of Reactive Oxygen Species Production in ABI1-1 and ABI2-1 Protein Phosphatase 2C Mutants.” *The Plant Cell* 13 (11): 2513–2523.

Nagy, Szilvia K., Zsuzsanna Darula, Brigitta M. Kállai, László Bögre, Gábor Bánhegyi, Katalin F. Medzihradszky, Gábor V. Horváth, and Tamás Mészáros. 2015. “Activation of AtMPK9 through Autophosphorylation That Makes It Independent of the Canonical MAPK Cascades.” *Biochemical Journal* 467 (1): 167–175.

Nishimura, Noriyuki, Ali Sarkeshik, Kazumasa Nito, Sang-Youl Park, Angela Wang, Paulo C. Carvalho, Stephen Lee, Daniel F. Caddell, Sean R. Cutler, and Joanne Chory. 2010. “PYR/PYL/RCAR Family Members Are Major in-vivo ABI1 Protein Phosphatase 2C-Interacting Proteins in Arabidopsis.” *The Plant Journal* 61 (2): 290–299.

Otterhag, Lotta, Marianne Sommarin, and Christophe Pical. 2001. “N-Terminal EF-Hand-like Domain Is Required for Phosphoinositide-Specific Phospholipase C Activity in Arabidopsis Thaliana.” *FEBS Letters* 497 (2–3): 165–170.

Pappan, Kirk L., and Xuemin Wang. 2013. “Assaying Different Types of Plant Phospholipase D Activities in Vitro.” In *Plant Lipid Signaling Protocols*, 205–217. Springer.

Park, Sang-Youl, Pauline Fung, Noriyuki Nishimura, Davin R. Jensen, Hiroaki Fujii, Yang Zhao, Shelley Lumba, Julia Santiago, Americo Rodrigues, and F. Chow Tsz-fung. 2009. “Abscisic Acid Inhibits Type 2C Protein Phosphatases via the PYR/PYL Family of START Proteins.” *Science* 324 (5930): 1068–1071.

Rubio, Silvia, Americo Rodrigues, Angela Saez, Marie B. Dizon, Alexander Galle, Tae-Houn Kim, Julia Santiago, Jaume Flexas, Julian I. Schroeder, and Pedro L. Rodriguez. 2009. “Triple Loss of Function of Protein Phosphatases Type 2C Leads to Partial Constitutive Response to Endogenous Abscisic Acid.” *Plant Physiology* 150 (3): 1345–1355.

Saez, Angela, Nadia Robert, Mohammad H. Maktabi, Julian I. Schroeder, Ramón Serrano, and Pedro L. Rodriguez. 2006. “Enhancement of Abscisic Acid Sensitivity and Reduction of Water Consumption in Arabidopsis by Combined Inactivation of the Protein Phosphatases Type 2C ABI1 and HAB1.” *Plant Physiology* 141 (4): 1389–1399.

Sanders, Dale, Jérôme Pelloux, Colin Brownlee, and Jeffrey F. Harper. 2002. “Calcium at the Crossroads of Signaling.” *The Plant Cell* 14 (suppl 1): S401–S417.

Sasaki, Takayuki, Izumi C. Mori, Takuya Furuichi, Shintaro Munemasa, Kiminori Toyooka, Ken Matsuoka, Yoshiyuki Murata, and Yoko Yamamoto. 2010. “Closing Plant Stomata Requires a Homolog of an Aluminum-Activated Malate Transporter.” *Plant and Cell Physiology* 51 (3): 354–365.

Scherzer, Sönke, Tobias Maierhofer, Khaled AS Al-Rasheid, Dietmar Geiger, and Rainer Hedrich. 2012. “Multiple Calcium-Dependent Kinases Modulate ABA-Activated Guard Cell Anion Channels.” *Molecular Plant* 5 (6): 1409–1412.

Schwartz, Amnon. 1985. “Role of Ca^2+^ and EGTA on Stomatal Movements in Commelina Communis L.” *Plant Physiology* 79 (4): 1003–1005.

Sokolovski, Sergei, and Michael R. Blatt. 2004. “Nitric Oxide Block of Outward-Rectifying K^+^ Channels Indicates Direct Control by Protein Nitrosylation in Guard Cells.” *Plant Physiology* 136 (4): 4275–4284.

Sridharamurthy, Madhuri, Amanda Kovach, Yang Zhao, Jian-Kang Zhu, H. Eric Xu, Kunchithapadam Swaminathan, and Karsten Melcher. 2014. “H_2_O_2_ Inhibits ABA-Signaling Protein Phosphatase HAB1.” *PLoS One* 9 (12): e113643.

Staxén, Irina, Christophe Pical, Lucy T. Montgomery, Julie E. Gray, Alistair M. Hetherington, and Martin R. McAinsh. 1999. “Abscisic Acid Induces Oscillations in Guard-Cell Cytosolic Free Calcium That Involve Phosphoinositide-Specific Phospholipase C.” *Proceedings of the National Academy of Sciences* 96 (4): 1779–1784.

Suhita, Dontamala, Agepati S. Raghavendra, June M. Kwak, and Alain Vavasseur. 2004. “Cytoplasmic Alkalization Precedes Reactive Oxygen Species Production during Methyl Jasmonate-and Abscisic Acid-Induced Stomatal Closure.” *Plant Physiology* 134 (4): 1536–1545.

Swatek, Kirby N., Rashaun S. Wilson, Nagib Ahsan, Rebecca L. Tritz, and Jay J. Thelen. 2014. “Multisite Phosphorylation of 14-3-3 Proteins by Calcium-Dependent Protein Kinases.” *Biochemical Journal* 459 (1): 15–25.

Tang, Ren-Jie, Hua Liu, Yang Yang, Lei Yang, Xiao-Shu Gao, Veder J. Garcia, Sheng Luan, and Hong-Xia Zhang. 2012. “Tonoplast Calcium Sensors CBL2 and CBL3 Control Plant Growth and Ion Homeostasis through Regulating V-ATPase Activity in Arabidopsis.” *Cell Research* 22 (12): 1650–1665.

Umezawa, Taishi, Naoyuki Sugiyama, Masahide Mizoguchi, Shimpei Hayashi, Fumiyoshi Myouga, Kazuko Yamaguchi-Shinozaki, Yasushi Ishihama, Takashi Hirayama, and Kazuo Shinozaki. 2009. “Type 2C Protein Phosphatases Directly Regulate Abscisic Acid-Activated Protein Kinases in Arabidopsis.” *Proceedings of the National Academy of Sciences* 106 (41): 17588–17593.

Wang, Xi-Qing, Hemayet Ullah, Alan M Jones, and Sarah M Assmann. 2001. “G Protein Regulation of Ion Channels and Abscisic Acid Signaling in Arabidopsis Guard Cells.” *Science* 292 (5524): 2070–2072.

Ward, John M., and Julian I. Schroeder. 1994. “Calcium-Activated K^+^ Channels and Calcium-Induced Calcium Release by Slow Vacuolar Ion Channels in Guard Cell Vacuoles Implicated in the Control of Stomatal Closure.” *The Plant Cell* 6 (5): 669–683.

Yu, Feng, Lichao Qian, Candida Nibau, Qiaohong Duan, Daniel Kita, Kathryn Levasseur, Xiaoqian Li, Changqing Lu, Hui Li, and Congcong Hou. 2012. “FERONIA Receptor Kinase Pathway Suppresses Abscisic Acid Signaling in Arabidopsis by Activating ABI2 Phosphatase.” *Proceedings of the National Academy of Sciences* 109 (36): 14693–14698.

Zhang, Wei, Liu-Min Fan, and Wei-Hua Wu. 2007. “Osmo-Sensitive and Stretch-Activated Calcium-Permeable Channels in *Vicia faba* Guard Cells Are Regulated by Actin Dynamics.” *Plant Physiology* 143 (3): 1140–1151.

Zhang, Wei, Byeong Wook Jeon, and Sarah M. Assmann. 2011. “Heterotrimeric G-Protein Regulation of ROS Signalling and Calcium Currents in Arabidopsis Guard Cells.” *Journal of Experimental Botany* 62 (7): 2371–2379.

Zhang, Wenhua, Chunbo Qin, Jian Zhao, and Xuemin Wang. 2004. “Phospholipase Dα1-Derived Phosphatidic Acid Interacts with ABI1 Phosphatase 2C and Regulates Abscisic Acid Signaling.” *Proceedings of the National Academy of Sciences* 101 (25): 9508–9513.

Zhang, Xiao, Hengbin Wang, Atsushi Takemiya, Chun-peng Song, Toshinori Kinoshita, and Ken-ichiro Shimazaki. 2004. “Inhibition of Blue Light-Dependent H^+^ Pumping by Abscisic Acid through Hydrogen Peroxide-Induced Dephosphorylation of the Plasma Membrane H^+^-ATPase in Guard Cell Protoplasts.” *Plant Physiology* 136 (4): 4150–4158.

Zhang, Yanyan, Huiying Zhu, Qun Zhang, Maoyin Li, Min Yan, Rong Wang, Liling Wang, Ruth Welti, Wenhua Zhang, and Xuemin Wang. 2009. “Phospholipase Dα1 and Phosphatidic Acid Regulate NADPH Oxidase Activity and Production of Reactive Oxygen Species in ABA-Mediated Stomatal Closure in Arabidopsis.” *The Plant Cell* 21 (8): 2357–2377.
